# Improving Signal Quality in Non-Contact Electrocardiography: Novel Strategy for Motion Artifact Reduction

**DOI:** 10.3390/s26123643

**Published:** 2026-06-07

**Authors:** Antonio Stanešić, Luka Klaić, Dino Cindrić, Mario Cifrek

**Affiliations:** University of Zagreb Faculty of Electrical Engineering and Computing, 10000 Zagreb, Croatia; luka.klaic@fer.unizg.hr (L.K.); dino.cindric@fer.unizg.hr (D.C.); mario.cifrek@fer.unizg.hr (M.C.)

**Keywords:** capacitive electrodes, noncontact electrodes, biopotentials, ECG, cECG, biomedical signals, gamification, NLMS, multi-referenced NLMS, QRS, PLI

## Abstract

Capacitive electrocardiography (cECG) enables non-contact heart rate monitoring through clothing, but motion artifacts remain a critical limitation for practical applications. We present a novel motion artifact removal method using non-contact floating electrodes as noise references combined with multi-reference Normalized Least Mean Squares (NLMS) adaptive filtering. The floating electrodes, positioned without skin contact, couple primarily to ambient 50 Hz mains interference, which becomes amplitude-modulated during motion due to changes in electrode–body capacitance. Six reference signals are derived from this noise electrode: band-pass-filtered signal and its derivative (capturing baseline-type artifacts), envelope and its derivative (capturing amplitude modulation patterns), and envelope asymmetry and its derivative (capturing non-linear electrode response during motion). The NLMS algorithm adaptively combines these references to estimate and remove motion artifacts while preserving QRS morphology through low-pass filtering of the correction signal. A hysteresis-based motion detector with minimum duration constraints enables selective application of artifact removal only during motion periods, leaving rest-period ECG unmodified. We present this as a proof-of-concept validation of a novel reference-electrode architecture for motion artifact suppression in non-contact ECG. The method was validated on 7 subjects across 24 recording sessions using two electrode configurations in two environments with different electromagnetic interference levels. Controlled axial rotation motion was induced at three frequencies using a custom apparatus with IMU-based gamification for protocol adherence. Performance was evaluated using R-peak detection *F1* score against gel surface-contact electrodes ground truth and RMS reduction in motion regions. Results demonstrate consistent improvement in R-peak detection accuracy during motion periods with substantial artifact energy reduction. The proposed method is designed to address motion artifacts regardless of their physical source, though the present validation focused on subject-induced motion.

## 1. Introduction

Ubiquitous healthcare is moving monitoring and decision-making beyond clinics into everyday life. Advances in wearable technologies allow continuous acquisition of biopotentials, capturing subtle physiological changes as people go about their routines. When paired with robust signal processing and data analysis, these measurements can reveal early signs of disease, track recovery, and personalize treatment in real time. This shift from episodic checkups to continuous insight has the potential to improve outcomes while reducing strain on traditional healthcare systems.

Electrocardiogram (ECG), as the recording of the heart’s electrical activity, is the most widely used and clinically informative biopotential signal for assessing cardiovascular health. Its non-invasive nature, relatively low acquisition cost and strong diagnostic value make it especially well suited for continuous monitoring in ubiquitous healthcare settings, where early detection of abnormalities and long-term trend analysis are essential. Beyond traditional clinical settings, ECG is increasingly used in everyday and non-medical contexts, such as wearable fitness devices, remote patient monitoring, and stress assessment [1,2,3,4]. In particular, analysis of R–R intervals enables the extraction of heart rate variability (HRV) metrics, which provide insight into autonomic nervous system functions and have demonstrated diagnostic and prognostic value in conditions such as cardiac dysfunction, stress-related disorders, and overall cardiovascular risk [5,6,7].

Traditionally, biopotential signals can be acquired using either hypodermic-contact needles or surface-contact electrodes, each suited to different applications. Hypodermic-contact needle electrodes provide highly localized and high-fidelity recordings by penetrating the skin and bypassing its filtering properties, making them valuable in clinical diagnostics and research where precise signal capture is required. In contrast, surface-contact electrodes are mostly non-invasive, more comfortable, and easier to deploy, which makes them preferable for long-term monitoring, wearable systems, and ubiquitous healthcare applications, despite their greater susceptibility to noise and motion artifacts.

Most common among surface-contact electrodes are traditional gel-based types, such as Ag/AgCl electrodes, which provide low skin–electrode impedance and high signal quality due to the presence of an electrolytic gel. However, their performance can degrade over time as the gel dries out, while prolonged use may cause skin irritation, making them less suitable for long-term or repeated monitoring. Dry surface-contact electrodes, on the other hand, eliminate the need for gel, improving ease of use, comfort, and suitability for wearable and long-duration applications, but they typically exhibit higher impedance and increased sensitivity to motion artifacts, which can compromise signal quality. Both gel (also called “wet”) and dry surface-contact electrodes are based on the direct contact with the skin of the subject.

To further address the limitations of such surface-contact electrodes, non-contact electrodes have been introduced, often referred to as capacitive electrodes due to the capacitive coupling mechanism they rely on (and the resulting ECGs are thus called capacitive ECGs or cECGs). These electrodes can acquire biopotential signals through coupling layers, such as clothing, offering superior comfort and eliminating issues related to skin preparation, irritation, and long-term wear. They also enable seamless integration into everyday objects, such as garments, mattresses, and seating, supporting unobtrusive and passive physiological monitoring [8,9,10]. However, the weak coupling capacitance makes them highly sensitive to motion, environmental noise, and parasitic capacitances, requiring high-input-impedance front-end circuits and advanced signal processing to achieve reliable measurements.

For consistency, in accordance with all the intricacies of non-contact electrodes and capacitive coupling [11], the term *non-contact electrodes* is adopted throughout this paper, although *capacitive electrodes* and *non-contact capacitive electrodes* are also terms commonly used in the literature. In addition, following common usage in the literature, the terms *cECG* and *non-contact ECG* are used interchangeably to denote ECG signals acquired using non-contact electrodes.

A typical non-contact ECG acquisition system based on capacitive coupling follows a layered signal chain designed to preserve weak biopotential signals. The non-contact electrode first forms a coupling capacitance with the body, through which the cardiac-induced electric field is sensed without direct skin contact. This signal is fed to a high-input-impedance preamplifier located as close as possible to the electrode to minimize signal attenuation and interference, after which an analog front-end (AFE) performs amplification, filtering, and common-mode noise suppression. The conditioned signal is then digitized by an analog-to-digital converter (ADC) and passed to subsequent digital processing stages, where artifact reduction, feature extraction, and higher-level analysis are performed. A more comprehensive treatment of non-contact electrodes and acquisition system design can be found in our previous studies [11,12,13,14,15,16,17].

Non-contact ECG electrodes introduce a range of signal integrity challenges, many of which overlap with those encountered in conventional surface-contact electrodes, such as motion-induced artifacts and mains interference. Power-line interference (PLI) is particularly prominent in both non-contact and contact systems, acting as a significant source of extrinsic noise. Motion artifacts, however, are widely regarded as the dominant limitation in non-contact ECG systems and are described as “the primary concern of capacitive measurement systems” in a recent comprehensive review [18]. The review further points out that motion artifacts in non-contact ECG recordings are broadly attributed to relative motion between the body and electrode, encompassing both physiological and voluntary movements. Although respiration-related baseline wander is sometimes treated separately, motion-induced disturbances are generally more severe and pose a greater challenge to robust ECG feature extraction.

Importantly, this prevailing view largely confines motion artifacts to subject- or electrode-induced effects. Motion in the surrounding environment, such as the motion of nearby people or conductive objects, can likewise disturb the ambient electric-field geometry and introduce artifacts, yet this mechanism is rarely treated explicitly in the cECG literature and is often subsumed under the broader non-stationary electromagnetic interference category or overlooked altogether. Nevertheless, such environmental motion artifacts are likely to be particularly common in clinical environments, where the routine movement of healthcare personnel, mobile medical equipment, and conductive infrastructure (e.g., nurses, metal carts, beds) can repeatedly disturb the ambient electric-field distribution.

Additional disturbances include triboelectric artifacts arising from friction between the body, clothing, and electrode surfaces (which can generate substantial static charge accumulation and induce large transient voltages), as well as cable microphonics resulting from mechanical movement of the leads. Furthermore, pressure applied to the sensing area can have a non-linear impact on the effective coupling area of the equivalent coupling capacitor, as demonstrated in our prior work [12]. All of these effects are exacerbated in non-contact measurement systems due to the inherently weaker signal coupling and higher source impedance compared to conventional gel surface-contact electrodes. As a result, repeatability of measurements is severely impaired. This issue was also investigated in depth in the same study.

To mitigate these disturbances, a variety of approaches have been proposed in the literature. PLI is commonly mitigated by employing high-performance instrumentation amplifiers, active guarding [19] and capacitive driven right leg (cDRL) circuits [13,20]. Flexible electrodes have been developed in order to maximize conformity with the body of the subject [21,22,23] and to minimize subject motion artifacts (via minimizing motion between the electrode and the body). Aside from those hardware and material approaches, many digital signal processing (DSP) and adaptive filtering methods have been developed, which can broadly be categorized into [18]:

1. Self-referencing techniques (such as template-based subtraction, wavelet denoising, empirical mode decomposition—EMD, etc.);
2. Reference-assisted techniques (such as adaptive filtering using inertial measurement units—IMU, electrode–tissue impedance—ETI, etc.).

Self-referencing approaches operate exclusively on the recorded ECG signal without relying on external reference measurements. Time–frequency techniques, such as wavelet denoising, have also been applied to non-contact ECG, offering effective separation of transient artifacts, although their performance is highly dependent on parameter selection and they are most often implemented in offline or semi-offline settings [24,25]. More advanced decomposition methods, including EMD and ensemble EMD (EEMD), can isolate motion-related components but are computationally demanding and inherently non-causal, which limits their suitability for real-time applications [26,27,28,29]. Blind source separation (BSS) techniques, such as principal component analysis (PCA) and independent component analysis (ICA), have likewise been explored [30,31,32,33,34,35]; however, they generally require multi-channel recordings and are predominantly applied in offline post-processing frameworks. Moreover, these methods rely on assumptions of source independence and spatially uniform noise across channels, which are often violated in non-contact ECG systems due to channel-dependent electrode–body coupling variations [18].

Reference-assisted approaches estimate motion artifacts using auxiliary signals that are correlated with the disturbance but ideally uncorrelated with the underlying cardiac activity. IMU/accelerometer-based methods exploit correlations between electrode or subject motion and ECG artifacts to enable adaptive filtering; however, these techniques are inherently limited to detecting motion of the sensor or subject and remain largely insensitive to environmental motion that perturbs the surrounding electric field [36,37]. More recently, impedance-based techniques (particularly those leveraging ETI as a reference) have been proposed to capture variations in the electrode–body interface associated with motion [36,38,39]. While such methods can improve artifact estimation under certain conditions, the relationship between ETI variations and motion artifacts is not universally strong or straightforward. Prior studies have shown that ETI exhibits the highest correlation with locally induced electrode artifacts, reduced correlation with skin deformation, and substantially weaker correlation with global body motion, limiting its effectiveness when the dominant artifact mechanisms are not directly reflected in impedance changes [18]. As a result, ETI-based approaches may not fully capture disturbances arising from external electric-field perturbations or more global motion patterns.

These approaches address specific classes of motion artifacts but do not offer a unified solution capable of simultaneously suppressing both subject-induced and environmentally induced motion artifacts in non-contact ECG systems. To address this gap, we propose a novel motion artifact suppression strategy for non-contact ECG that is designed to be causal and composed exclusively of operations compatible with real-time implementation. In this work, the method is implemented in MATLAB (R2025b; MathWorks^®^: Natick, MA, USA, 2025) to facilitate systematic validation, while real-time deployment is reserved for future investigation. The present study is a proof-of-concept validation of this architecture under controlled conditions, with population-level evaluation deferred to future work.

During our prior investigations into non-contact electrodes [12,13,14], we observed that both classical motion artifacts (arising from movement of the electrodes or subject) and environmental motion artifacts (arising from movement of nearby persons or conductive objects) are registered by non-contact electrodes even when those electrodes are off-body and left floating. This observation motivates a double electrode pair approach: one pair (also called the primary pair, and their corresponding signal “primary signal”) serves as the signal electrodes for cECG acquisition, while a second pair (also called the secondary pair, and their corresponding signal “secondary signal”), intentionally left floating, serves as a noise reference that captures motion-induced disturbances without ECG content. By deriving reference signals from these noise electrodes and applying adaptive filtering, artifacts correlated between the two pairs can be suppressed regardless of their source. Related concepts exploiting auxiliary electrodes or reference signals have been explored previously [40,41,42,43], particularly in the context of interference estimation; however, the proposed configuration and acquisition protocol in this research are specifically designed to capture both subject-induced and environmentally induced motion artifacts, as well as advance the general concept one step further.

The rest of the paper is organized as follows: Section 2 will detail the hardware configuration, reference signal derivation, and adaptive filtering methodology. Section 3 delivers obtained results, along with performance evaluation and method validation, while Section 4 presents the discussion of the results and positions them in the broader context of the existing research. Lastly, Section 5 delivers concluding remarks. A list of abbreviations used throughout the paper is then provided for reference. The appendices present additional technical details, including the full algorithm pseudocode, screenshots of the gamification interface, and the full result table.

The main contributions of this work are:

- A cECG setup with a double electrode pair in which a dedicated floating noise electrode pair captures motion-induced disturbances without cardiac content, providing artifact references that are agnostic to the physical source of the disturbance;
- Six complementary reference signals from this single noise channel derived from the noise electrode pair, including a novel envelope asymmetry reference that exploits non-linear capacitive coupling effects not previously described in the context of motion artifact removal;
- Integrating these references into a multi-reference NLMS adaptive filtering pipeline with motion-gated correction and QRS-preserving mechanisms, all composed of causal operations suitable for real-time implementation;
- Validation of the method across seven subjects, two electrode configurations, and two electromagnetic environments using a gamification-based protocol for standardized motion induction.

## 2. Materials and Methods

### 2.1. System Overview

**Figure 1:**
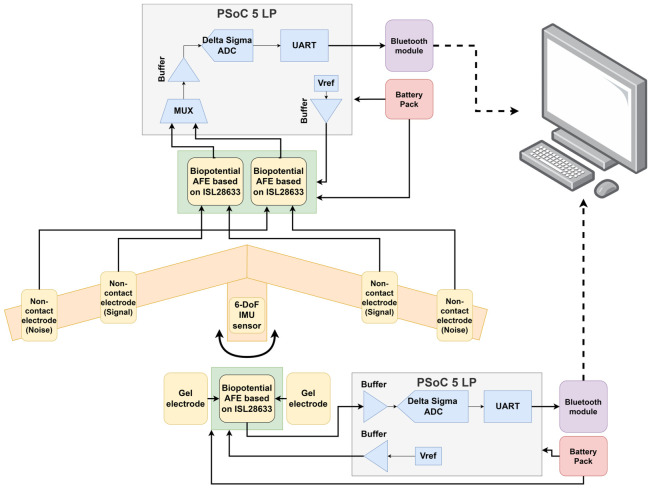
Block diagram of the experimental measurement system comprising two galvanically isolated, battery-powered acquisition units. Non-contact electrodes are positioned in the coplanar configuration, as discussed in the following subsection.

The proposed method employs a double pair of electrodes for non-contact ECG acquisition with integrated motion artifact reference. As illustrated in Figure 1, the system comprises two functionally distinct electrode pairs:

1. Signal electrodes, positioned to acquire the ECG (primary pair);
2. Noise electrodes, intentionally placed to capture motion-induced disturbances without significant ECG content (secondary pair).

Both electrode pairs feed into identical AFE circuits, implemented on the same printed circuit board (PCB) and connected to the same ADC via a multiplexer, ensuring matched frequency response and timing characteristics. A conventional gel electrode ECG, using the same AFE and ADC, serves as the gold standard for validation. The digitized signals are transmitted wirelessly via Bluetooth to a host computer for processing, and the entire acquisition setup is battery-powered to ensure galvanic isolation.

The signal processing pipeline consists of three main stages. First, six reference signals are derived from the noise electrode channel, each designed to capture different aspects of motion-induced interference, ranging from direct baseline shifts to non-linear amplitude modulation of the mains coupling. Second, an ECG-based motion detector identifies time intervals where artifact correction is required. Third, a multi-reference normalized least mean squares (NLMS) adaptive filter uses the derived references to estimate and remove artifacts from the ECG signal, with provisions to prevent overcorrection and preserve QRS morphology.

The electrodes are mounted on a wooden axial rotation apparatus, serving as an emulator of the steering wheel, equipped with an IMU which provides six degrees of freedom tracking (6-DoF). The IMU enables a gamification-based protocol in which subjects track a visual target by rotating the apparatus, facilitating controlled and repeatable motion patterns across subjects and sessions. All signals are acquired synchronously to enable direct comparison. The following subsections describe each system component in detail: the electrodes and electrode configurations (Section 2.2), analog front-end and data acquisition (Section 2.3), reference signal derivation (Section 2.4), adaptive filtering algorithm (Section 2.5), and experimental protocol (Section 2.6).

### 2.2. Electrodes and Electrode Configurations

**Figure 2:**
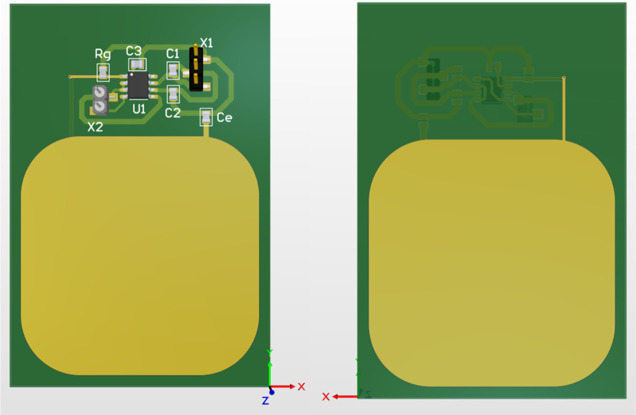
3D model of non-contact electrodes used in the research, front (**left**) and back (**right**) view. More details are available in our prior work [12,14].

In this study, we have used the non-contact electrodes used in our prior research [12,14] for both pairs of electrodes (primary and secondary—signal and noise). The electrodes are shown in Figure 2. Instead of the original rectangular sensor surface described in [44], electrodes were designed with rounded rectangular sensor surfaces to mitigate corner-induced electric-field concentration, which is known to contribute to edge effects and elevated noise levels [45]. Electrodes are based on a precision LMP7721 amplifier (Texas Instruments, Dallas, TX, USA). Shielded cables were employed to isolate the output signals from environmental noise, and the solder mask was removed from the sensing area to avoid the introduction of another dielectric and minimize surface leakage. Each of the electrodes has a sensing area of approx. 20 cm^2^ and is manufactured in typical FR4 two-layer rigid PCB technology.

**Figure 3:**
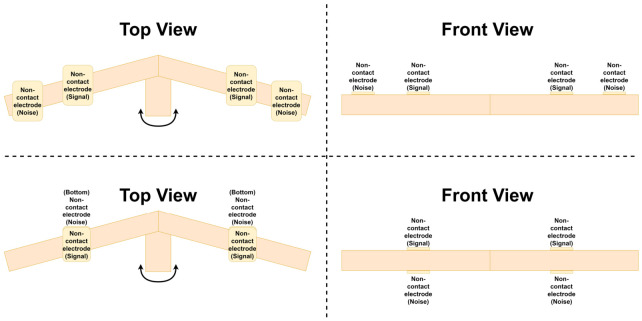
Illustration of the evaluated electrode configurations. (**Upper left**)—Top View of coplanar configuration; (**Upper right**)—Front view of coplanar configuration; (**Lower left**)—Top view of stacked configuration; (**Lower right**)—Front view of stacked configuration.

Two electrode configurations were evaluated in this study, selected to represent distinct geometric arrangements of the signal and noise electrode pairs (Figure 3). In the coplanar configuration, both pairs are mounted on the same plane (thus coplanar), with the noise electrode pair positioned laterally outward from the signal electrode pair. This arrangement exposes the noise electrodes to a larger effective area of the ambient electric field, resulting in greater 50 Hz pickup and, consequently, stronger envelope and asymmetry reference signals. In the stacked configuration, the signal electrode pair is mounted on the upper surface of the wooden handle while the noise electrode pair is positioned directly beneath on the lower surface of the handle. This parallel-plane arrangement offers a more compact form factor while maintaining similar motion coupling between the electrode pairs, as both experience the same mechanical displacement during movement. In both configurations, signal and noise electrode pairs utilize identical electrode geometry and AFE circuitry. Because the noise electrodes are not in close proximity to the body, their coupling to biopotentials is negligible; instead, they couple predominantly to the ambient electric field, which is dominated by 50 Hz mains interference in typical indoor environments. Details on the measurement setup are given in Section 2.6.

### 2.3. Analog Front-End and Data Acquisition

The analog front-end used for interfacing both pairs of non-contact electrodes has been introduced in our prior work [12,14]. However, due to the nature of this research, one PCB contains two matched AFEs: one used for the signal, and another for the noise pair of electrodes. This approach was adopted to reduce mismatches arising from variations in circuit topology and to facilitate easier cable management. Since the PCB is interfacing towards a single microcontroller board, this enables simultaneous and exact gain switching in the instrumentation amplifier stage, if necessary.

Each AFE branch consists of an input differential low-pass filter, which serves as both a radio frequency interference (RFI) filter and an anti-aliasing filter (AAF) with a corner frequency of 500 Hz, and an ISL28633 (Renesas Electronics, Tokyo, Japan) fully differential instrumentation amplifier [46] with configurable gain via tri-state pins. Both branches are connected to a PSoC 5LP (Infineon Technologies, Neubiberg, Germany) microcontroller [47] that uses an internal multiplexer component to multiplex two analog signals and an internal Delta-Sigma ADC for signal digitization. Each channel is sampled at *f*_s_ (1000 Hz) with 16-bit resolution. A $V_{ref}=1.024\;V$ reference voltage, generated, internally bypassed, and buffered within the microcontroller, is used for all electrodes. Given the ADC differential input range of $\pm 2\;V_{ref}$ and a total AFE gain of 4, the input-referred conversion factor is $V_{in}=code\;\left[a.u.\right]*\frac{V_{ref}}{2^{16}}$. This corresponds to 15.625 µV/count (input-referred), with an input-referred full-scale range of ±0.512 V.

### 2.4. Reference Signal Derivation

**Figure 4:**
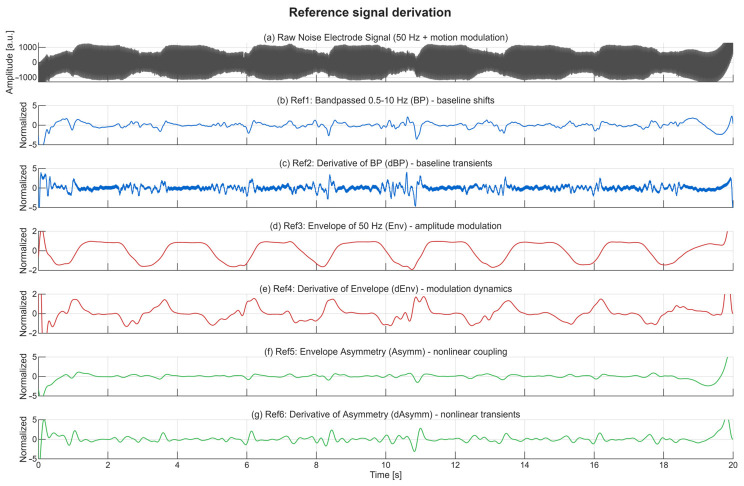
Complete set of reference signals derived from secondary pair of noise electrodes for multi-reference adaptive filtering (Subject 2, coplanar configuration, Environment A). (**a**) Raw signal dominated by 50 Hz mains coupling with visible motion-induced amplitude modulation. (**b**,**c**) Band-pass-derived references: The 0.5–10 Hz band-passed signal (BP) captures low-frequency baseline shifts, while its derivative (dBP) emphasizes rapid baseline transients. (**d**,**e**) Envelope-derived references: The mean envelope magnitude (Env) tracks amplitude modulation of the mains interference, and its derivative (dEnv) captures modulation dynamics. (**f**,**g**) Asymmetry-derived references: The envelope asymmetry signal (Asymm) reflects non-linear coupling differences between positive and negative interference half-cycles, with its derivative (dAsymm) highlighting non-linear transient components. All reference signals are normalized and zero-meaned for input to the NLMS adaptive filter.

A total of six reference signals were derived from the noise non-contact electrode pair for usage in the adaptive filtering algorithm. Each of the reference signals has been designed to capture different characteristics of motion-induced interference. These references can be grouped into three categories based on the underlying physical mechanism they exploit. Illustration of all six references is shown in Figure 4.

Baseline-type references are obtained by band-pass filtering the noise electrode signal (0.5–10 Hz, Butterworth 2nd order), capturing direct motion-induced baseline shifts that occur when movement alters the electrode–body capacitance. The first reference (BP) represents these slow baseline variations, while its derivative (dBP) captures transient components associated with abrupt movements.

Envelope-type references exploit the amplitude modulation of the 50 Hz mains coupling caused by motion. As the electrode–body distance or orientation changes, the capacitive coupling to the ambient electric field is modulated, causing the amplitude of the 50 Hz interference to vary. The envelope is extracted by rectifying the noise signal and low-pass filtering with a 4 Hz cutoff frequency (Butterworth, 4th order). The envelope signal (Env) captures slow amplitude variations, while its derivative (dEnv) captures rapid modulation dynamics.

**Figure 5:**
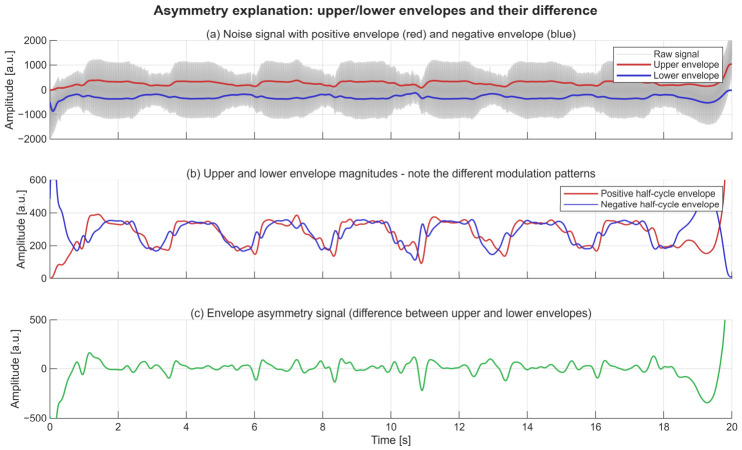
Derivation of envelope-based reference signals from secondary pair of electrodes (Subject 2, coplanar configuration, Environment A). (**a**) Raw signal from a non-contact electrode showing dominant 50 Hz mains coupling, with upper (red) and lower (blue) envelopes extracted via peak detection. (**b**) Magnitudes of positive and negative half-cycle envelopes, revealing distinct amplitude modulation patterns induced by motion affecting electrode–skin coupling. (**c**) Envelope asymmetry signal computed as the difference between upper and lower envelope magnitudes, capturing motion-induced variations in the capacitive coupling that manifest differently for positive and negative interference half-cycles.

Asymmetry-type references capture non-linear aspects of the capacitive coupling that symmetric envelope detection cannot reveal. During each 50 Hz cycle, the positive and negative half-cycles may be affected differently by motion due to the non-linear nature of the electric-field distribution around the electrode. This asymmetry is quantified by separately extracting the envelopes of the positive and negative half-cycles and computing their difference. Figure 5 illustrates this phenomenon: the upper and lower envelopes of the noise signal exhibit visibly different modulation patterns during motion. The asymmetry signal (Asymm) and its derivative (dAsymm) provide reference signals that complement the symmetric envelope, particularly for fast or non-linear motion artifacts.

**Figure 6:**
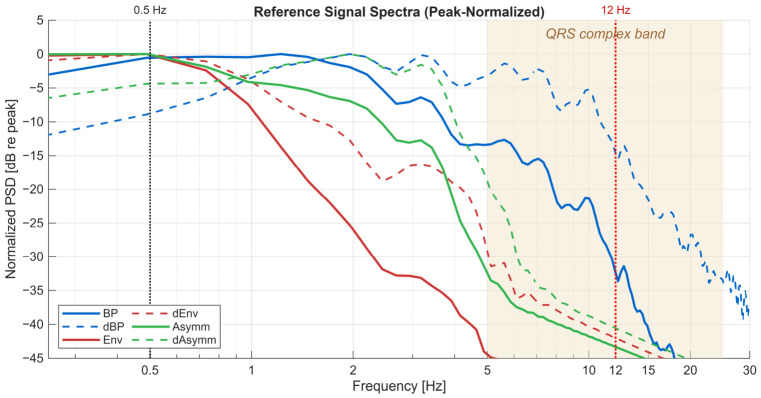
Power spectral density (PSD) of the six reference signals derived from the secondary pair of electrodes (Subject 2, coplanar configuration, Environment A), peak-normalized to facilitate spectral shape comparison (dB re peak: dB relative to peak). The shaded region indicates the QRS complex band (5–15 Hz); the 12 Hz correction signal low-pass filter cutoff (vertical red dashed line) is chosen to enable artifact cancelation while preserving R-peak morphology.

Power spectral density (PSD) of the six reference signals derived from secondary pair of electrodes, peak-normalized to facilitate spectral shape comparison, is shown in Figure 6. The band-pass-derived references (BP, dBP) exhibit the broadest spectral content, extending into the QRS complex band (shaded region, 5–15 Hz). Envelope-based references (Env, dEnv) are predominantly low frequency. Asymmetry-derived references (Asymm, dAsymm) occupy an intermediate spectral range, capturing non-linear coupling dynamics. The 12 Hz cutoff frequency for the NLMS correction signal low-pass filter (vertical red dashed line) is selected to permit artifact cancelation up to the lower edge of the QRS band while attenuating filter output in the frequency range critical for R-peak preservation. The 0.5 Hz marker indicates the lower bound of the noise pair passband. These spectral characteristics enable the multi-reference approach to address artifacts across different frequency ranges, targeting slow baseline drift with envelope references and addressing faster motion components with band-pass and asymmetry references.

**Figure 7:**
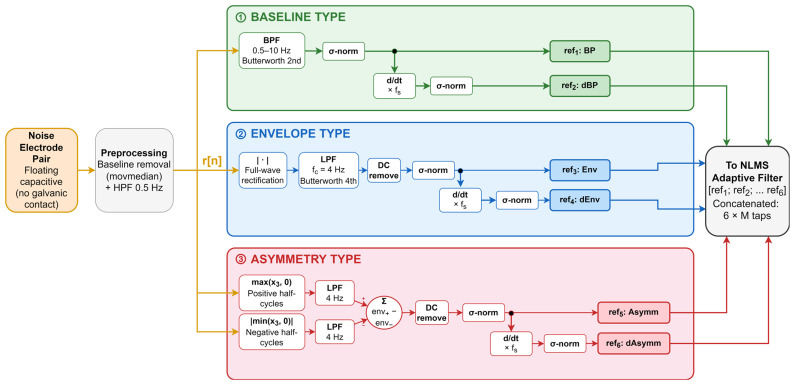
Signal processing pipeline for deriving six reference signals from the floating noise electrode pair. All references are direct-current-removed (DC-removed) and σ-normalized before concatenation into a 6 × M tap input vector for the NLMS adaptive filter, where M denotes filter order.

All six references are normalized to unit standard deviation prior to adaptive filtering. Multi-reference approach ensures that various artifact types can be captured by at least one reference signal, enabling the adaptive filter to address a broad range of motion artifact characteristics. Although the six reference signals are derived from a common source, they capture largely complementary aspects of the motion-induced interference. In particular, the derivative references (dBP, dEnv, dAsymm) exhibit low correlation with their non-derivative counterparts, as they emphasize transient dynamics rather than absolute variations. Similarly, the asymmetry references capture non-linear modulation effects that are largely orthogonal to the symmetric envelope. This diversity enables the adaptive filter to address a broad range of artifact characteristics without requiring manual selection of the most appropriate reference for each condition. The signal processing pipeline for deriving six reference signals from the secondary electrode pair is shown in Figure 7.

### 2.5. Adaptive Filtering Algorithm

The core of the motion artifact removal is the standard NLMS adaptive filtering algorithm. NLMS was selected for its computational efficiency, numerical stability and suitability for real-time implementation in embedded systems, aligning with the goal of developing a practical non-contact monitoring device. The normalized approach was chosen to avoid manual gain tuning, which would be required as signal levels change during motion artifacts. NLMS is also extensively validated for usage in ECG artifact removal [48,49].

**Figure 8:**
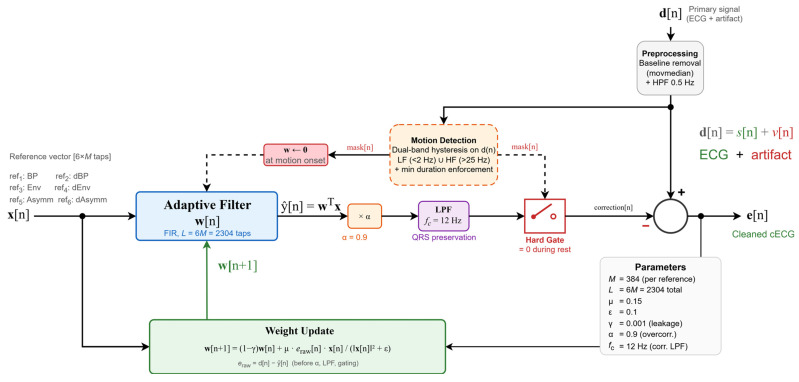
Block diagram of the multi-reference NLMS adaptive filtering system. The primary signal d[n], containing both ECG and artifacts, is preprocessed while the concatenated reference vector x[n] comprising six derived references (BP, dBP, Env, dEnv, Asymm, dAsymm) with *M* = 384 taps each feeds the adaptive filter (L = 6*M* = 2304 total taps). The filter output ŷ(n) is scaled by *α* = 0.9 to prevent overcorrection, low-pass filtered at 12 Hz for QRS preservation, and gated by the motion detection mask before subtraction from the primary signal. Motion detection operates on d[n] using dual-band monitoring (LF < 2 Hz, HF > 25 Hz) with hysteresis thresholds and minimum duration enforcement. Filter weights are reset at motion onset and updated via leaky NLMS (*γ* = 0.001) using the raw error signal computed before scaling, filtering, and gating, ensuring adaptation based on true residual error during motion segments only.

The adaptive filter operates by estimating the artifact component present in the primary (cECG) signal using one or more reference signals that are correlated with the motion artifact but not with the actual cardiac signal. The primary signal is modeled as:

(1) d[n]=s[n]+v[n]

where d[n] denotes the measured cECG signal on the primary pair of electrodes, s[n] the underlying cardiac component, and v[n] the artifact component. The filter coefficients are updated iteratively to minimize the error between the primary signal and the estimated artifact, yielding a cleaned output signal. Table 1 summarizes the algorithm parameters used in this study. A block diagram of the designed multi-reference NLMS adaptive filtering system is shown in Figure 8. It will be described in detail in the rest of this section.

Rather than selecting a single optimal reference, a multi-reference NLMS approach was implemented, wherein each reference type is processed by a dedicated adaptive filter and the outputs are combined. This strategy accommodates the varying artifact characteristics across motion frequencies and electrode configurations. The entire algorithm operates in three phases: motion detection, adaptive weight estimation, and correction application. The pseudocode is available in Appendix A.

Adaptive filtering is applied selectively to signal segments exhibiting motion artifacts, preserving the original signal during stationary periods. Motion detection is performed directly on the ECG signal rather than on the noise electrode channel, ensuring that correction is applied only when artifacts are actually present in the signal of interest. This ECG-based approach is necessary due to the fact that the content of artifacts visible to the noise electrode pair is a superset of artifacts visible to the signal electrode pair. Thus, they may register environmental interference that does not manifest as an artifact in the signal electrodes.

Motion detection is based on two short-time variance (STV) metrics computed from the ECG using fully overlapping sliding windows. A low-frequency STV, obtained from a baseline estimate of the ECG (<2 Hz), captures baseline wander, while a high-frequency STV, obtained from a high-pass filtered ECG (>25 Hz), captures transient disturbances outside the typical QRS frequency band. Variance is estimated using a sliding-window sample variance, with window lengths of 1 s and 2 s for the low- and high-frequency branches, respectively. For a signal x[n] and window length W, the sliding-window mean $\bar{x}\left[n\right]$ and variance $\sigma_{x}^{2}\left[n\right]$ are defined as:

(2) $$\bar{x}\left[n\right]=\frac{1}{W}\sum_{k=n-W+1}^{n}x\left[k\right]$$

(3) $$\sigma_{x}^{2}\left[n\right]=\frac{1}{W-1}\sum_{k=n-W+1}^{n}{\left(x\left[k\right]-\bar{x}\left[n\right]\right)}^{2}$$

Each metric is normalized by a robust (insensitive to outliers) percentile of its empirical distribution and evaluated using fixed hysteresis thresholds with separate entry and exit levels. Motion is detected when either metric exceeds its threshold, and the combined detection mask is subsequently regularized using minimum motion and rest duration constraints to reduce rapid state oscillation between motion and rest states.

The six reference signals are stacked into a single 6*M*-dimensional reference vector $x[n]\in R^{6M}$, containing *M* delayed samples for each reference. During detected motion intervals, the six reference signals are concatenated into a single reference vector and processed by an NLMS adaptive filter. The filter estimates a set of weights that, when applied to the reference vector, produce an estimate of the artifact component present in the ECG signal, as follows:

(4) $$\hat{v}[n]=w^{⊤}[n]\;x[n]$$

where w[n] denotes the adaptive weight vector. This estimate is subtracted from the ECG signal to produce the cleaned output. The instantaneous error used for adaptation is defined as:

(5) $$e[n]=d[n]-w^{⊤}[n]\;x[n]$$

which corresponds to the residual signal after subtracting the unscaled artifact estimate. The weights are then updated according to the NLMS update rule, which normalizes the adaptation step by the reference signal energy to ensure stable convergence regardless of signal amplitude variations.

The filter order *M*, expressed in samples, determines the temporal span of the reference signal history available to the filter. A sufficiently large filter order allows the algorithm to compensate for variable delays between the reference signals and the ECG artifacts without requiring explicit lag estimation or alignment. For the sampling rate and artifact dynamics encountered in this study, a filter order (*M*) of 256–384 samples (256–384 ms for *f*_s_ = 1000 Hz) was found to provide adequate temporal coverage.

A known challenge with adaptive noise cancelation is overcorrection, which can occur when the reference signal contains components correlated with the desired signal or when the reference captures a superset of the interference present in the primary signal. Since the noise electrodes are more exposed to environmental disturbances than the signal electrodes, the derived references may contain interference components that do not appear in the ECG channel, potentially leading to signal distortion if the filter attempts to remove them.

Three mechanisms are employed to mitigate overcorrection. First, a leakage factor γ is incorporated into the weight update, causing weights to decay toward zero in the absence of consistent correlation. This prevents weight accumulation from spurious correlations and improves tracking of non-stationary artifacts. The leaky NLMS update rule is given by:

(6) $$w[n+1]=\left(1-\gamma \right)w[n]+\mu \frac{e[n]x[n]}{|x[n]{|}^{2}+\varepsilon }$$

where μ is the step size, γ is the leakage factor, and ε prevents numerical instability.

Second, a partial correction factor α scales the filter output before subtraction, limiting the maximum correction magnitude. Third, the correction signal is low-pass filtered prior to subtraction to attenuate components in the QRS frequency band, preserving cardiac waveform morphology at the expense of reduced artifact suppression at higher frequencies. The final cleaned ECG signal is therefore computed as:

(7) $$\hat{s}[n]=d[n]-\alpha \;g[n]\;L\{\hat{v}[n]\}$$

where α is the partial correction factor, g[n]∈{0,1} is the motion detector mask, and L{·} denotes low-pass filtering.

At each detected motion onset, filter weights are reset to zero, allowing fresh adaptation to the characteristics of the new artifact episode. This prevents inappropriate correction based on weights learned from previous, potentially different, artifact conditions.

### 2.6. Experimental Protocol

Both electrode pairs are mounted on a custom wooden apparatus designed to enable controlled and repeatable motion artifact induction. Wood was selected as the construction material due to its electrically insulating properties, ensuring that the apparatus itself does not introduce conductive pathways or alter the electric-field geometry around the electrodes. The apparatus permits axial rotation resembling a bicycle steering wheel motion, providing a natural and intuitive movement pattern for subjects while constraining motion to a single degree of freedom (nearly all of the motion is in yaw). This simplification facilitates reproducible artifact generation across subjects and sessions. Additionally, the rigid mounting of both electrode pairs on the same structure ensures that signal and noise electrodes experience identical mechanical displacement during rotation. Cabling from both electrode pairs is routed together along the apparatus, ensuring that any cable microphonics affects both channels similarly and can thus be rejected by the adaptive filter as common-mode interference.

**Figure 9:**
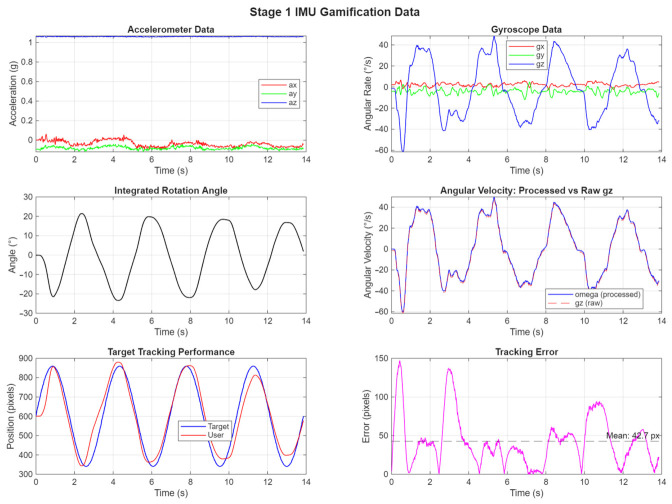
Example of data interpretation from Stage 1 of the gamification protocol for standardized motion artifact generation using IMU-based real-time feedback. (**Top row**): Raw accelerometer and gyroscope data from the apparatus-mounted IMU during controlled axial rotation, showing dominant motion in the *z*-axis (rotation about the vertical axis). (**Middle row**): Integrated rotation angle derived from gyroscope data, demonstrating consistent ±20° oscillation amplitude, alongside comparison of processed angular velocity with raw gyroscope output. (**Bottom row**): Target tracking performance from the PyGame (version 2.6.1; Pygame Project) visual interface, where subjects follow a sinusoidally moving target (blue) by rotating the apparatus; tracking error (right) shows a mean deviation of 42.7 pixels, indicating adequate adherence to the prescribed motion pattern.

To further enhance measurement repeatability and standardize motion patterns across subjects, a gamification-based protocol was implemented (shown in Appendix B, written in PyGame, version 2.6.1; Pygame Project). The apparatus is equipped with a 6-DoF IMU that tracks its orientation in real time. During measurement sessions, subjects view a display showing a moving target circle that oscillates horizontally at a specified frequency. Subjects are instructed to track the target by rotating the apparatus, with their current position indicated by a cursor driven by the IMU orientation data. This approach transforms the motion artifact induction task into an intuitive tracking exercise, reducing inter-subject variability in movement amplitude and timing while maintaining subject engagement throughout the session. Three target frequencies were selected for evaluation: 0.2 Hz representing slow, smooth movements (Stage 1); 0.4 Hz representing moderate movements (Stage 2); and 0.8 Hz representing fast, abrupt movements that approach or exceed typical real-world motion scenarios (Stage 3). Each measurement session followed a structured protocol alternating between stationary periods and motion periods at each frequency, enabling systematic comparison of artifact characteristics and removal performance across different motion dynamics. Motion intervals were 20 s long, with rest times dependent on the re-calibration of the rotation angles. Example of the processed IMU data for one stage (one motion interval, in this case Stage 1—0.2 Hz) is shown in Figure 9.

Our prior research into temporal stability of non-contact electrodes [12] established that an initial waiting period is necessary before obtaining comparable measurements. During this period, the signal-to-noise ratio (SNR) gradually stabilizes as the electrode–dielectric–skin interface reaches equilibrium. This stabilization typically requires about 10 min depending on subject body composition and skin characteristics and environmental conditions [12]. Consequently, the measurement protocol must accommodate this waiting period initially, and relevant measurements start after the SNR has stabilized.

A pair of gel surface-contact electrodes were used to record the reference signal simultaneously to provide ground-truth ECG morphology for quantitative evaluation of the adaptive filtering performance. This reference enables direct comparison of the filtered non-contact ECG against clinical-grade measurement, allowing assessment of both noise suppression effectiveness and preservation of diagnostically relevant features. It uses an identical AFE design (instrumentation amplifier, filtering stages, and ADC) to ensure that any differences between the reference and non-contact signals can be attributed solely to the electrode–skin interface rather than to downstream signal conditioning. Gel surface-contact electrodes used in this study were standard reusable clamp-type ECG electrodes with electrolytic conductive gel. These electrodes were selected based on their superior performance and signal stability under motion artifacts.

Crucially, both systems (the pair of reference gel-surface contact electrodes and the dual pair of non-contact electrodes) were galvanically isolated from mains power to ensure that the recorded motion artifacts are representative of standalone non-contact monitoring conditions. A mains-connected reference would alter the subject’s body potential dynamics, potentially introducing spurious interference or masking the capacitive coupling variations under study.

Galvanic isolation between the two measurement systems introduced two synchronization challenges. First, independent clock domains lead to sampling frequency disparity—even small differences in oscillator frequency accumulate over extended recordings, causing progressive temporal drift between channels. Second, Bluetooth connection establishment is inherently variable in timing, making it difficult to align the start of recordings without a shared trigger mechanism.

The first challenge was addressed by equipping both systems with precision external crystal oscillators (±20 ppm tolerance). While the systems may remain connected during the initial stabilization period of about 10 min, the analyzed measurement segments are typically up to 3 min in duration, bounding worst-case clock drift to under 4 ms. This is negligible relative to cardiac event timescales and readily correctable through resampling. If necessary, acquisition can be restarted after stabilization to reset synchronization, though in practice the achieved drift was sufficiently small that this was not required.

The second challenge was solved through a custom Python (Python 3.10; Python Software Foundation: Wilmington, DE, USA, 2021) acquisition application that manages both Bluetooth serial port profile (SPP) connections and initiates recording only after both channels have successfully connected and stabilized. This approach ensures sample-aligned recording starts without relying on post hoc cross-correlation alignment, which could be confounded by the very motion artifacts under study.

Each measurement is performed according to the following protocol:

1. The subject is informed about the measurement procedure, reasons for collecting each of the recorded data and the entire context of the measurement, and gives consent if they agree with presented conditions.
2. The subject is seated in the seat, and thin cotton gloves are placed on the subject’s hands.
3. The skin on the subject’s arms, where the gel surface-contact electrodes will be placed, is cleaned and prepared for the adhesion of gel surface-contact electrodes according to the common practice for such measurements.
4. Gel surface-contact electrodes are placed on each of the lower arms of the subject.
5. The subject places their hands over the sensing (primary) non-contact electrode pair. In the stacked configuration, the subject is instructed not to touch the secondary pair of electrodes with their fingers, while this separation is inherent with the topology of the coplanar configuration. This ensures that there is no risk of ECG signal leakage into the noise electrodes.
6. The signal is monitored until SNR stabilizes, which is confirmed by the measurement assistant.
7. The measurement assistant initiates the custom Python acquisition application, which establishes Bluetooth connections to both sensor systems and begins synchronized recording only once both channels are confirmed active. The assistant moves at least 2 m away from the subject to avoid capacitive coupling to the electrodes and introducing environmental motion artifacts. Once the assistant is positioned, the gamification protocol is started, guiding the subject through standardized motion sequences at progressively increasing frequencies via visual feedback displayed on a screen.
8. After the gamification protocol is over, the measurement is completed.

The protocol described was carried out for each of the two electrode configurations, coplanar and stacked, as described in Figure 3. In addition, to evaluate the method in presence of stronger and weaker PLI, two environments were tested: environment with greater PLI influence (Environment A, Env A), and environment with weaker PLI influence (Environment B, Env B). Thus, in total, four independent measurement sessions were performed per subject.

Both sensor systems transmitted data to a laptop via Bluetooth SPP, with each system assigned a dedicated communication (COM) port (communicating via a Universal Asynchronous Receiver-Transmitter, UART). A custom Python application managed the simultaneous connections and ensured a synchronized recording start. All ECG signals, including both the gel surface-contact electrode reference and non-contact electrode channels, were sampled at $f_{s}$ (1000 Hz). IMU data from the motion tracking sensor was recorded at 200 Hz for potential diagnostic use, such as verifying motion protocol compliance or correlating artifact severity with physical displacement. Post-acquisition signal processing and analysis were performed in the MATLAB (R2025b; MathWorks^®^: Natick, MA, USA, 2025) environment.

In total, measurements have been performed on 7 healthy, willing subjects (S1–S7). The demographic characteristics of the subjects, including age, sex, and body mass index (BMI), are summarized in Table 2. One of the subjects (S4) has a condition of semi-voluntary palmar hyperhidrosis, while other participants have no declared history of excessive hand sweating or related dermatological conditions which could influence measurement quality through the “hidden parameters” investigated in [12].

## 3. Results

### 3.1. Full Processing Pipeline Visualization

This section presents representative results from our extended measurement sessions to demonstrate the data processing workflow and analytical framework. Results from a single, representative session will be visualized to emphasize processing stages. This detailed analysis of an individual measurement establishes a basis for understanding the full collection of results summarized in Table 3, in Section 3.2.

**Figure 10:**
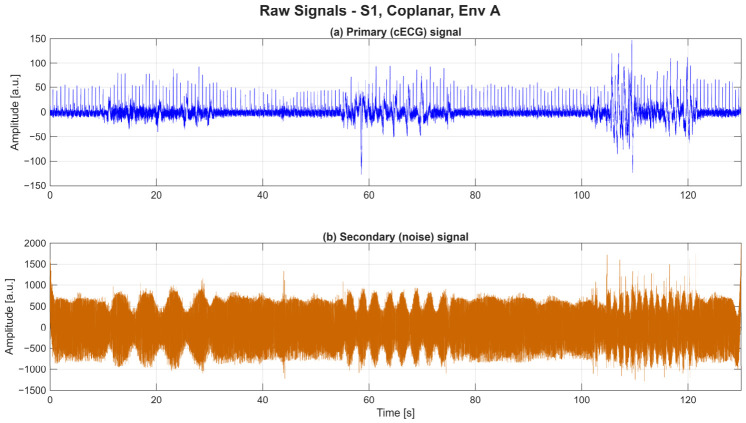
Raw signals from two pairs of non-contact electrodes (Subject 1, coplanar configuration, Environment A). (**a**) Primary signal (from the signal pair of non-contact electrodes) containing the target cECG, contaminated with motion artifacts (10–30 s for the “slow” motion, Stage 1, 55–75 s for the “medium” motion speed, Stage 2, and 105–125 s for the “fast” motion speed, Stage 3). (**b**) Secondary signal (noise reference from the noise pair of non-contact electrodes), dominated by mains coupling with minimal cardiac contribution due to its floating configuration.

Signals from the double pair of electrodes (primary and secondary pair) are shown in Figure 10. Significant artifacts are observable in motion segments (10–30 s for the “slow” motion, Stage 1, 55–75 s for the “medium” motion speed, Stage 2, and 105–125 s for the “fast” motion speed, Stage 3) in both primary and secondary signals. However, the secondary signal contains a significant amount of 50 Hz PLI not contained in the primary signal. This presents a problem if the secondary signal was to be used directly as a reference for NLMS algorithm. Notably, the secondary signal contains no discernible cardiac component, confirming that the floating electrode configuration couples primarily to environmental interference rather than biopotentials. Moreover, the interference captured by the secondary electrodes represents a superset of the noise present in the primary signal, encompassing both the motion-induced artifacts common to both channels and the additional 50 Hz mains coupling.

**Figure 11:**
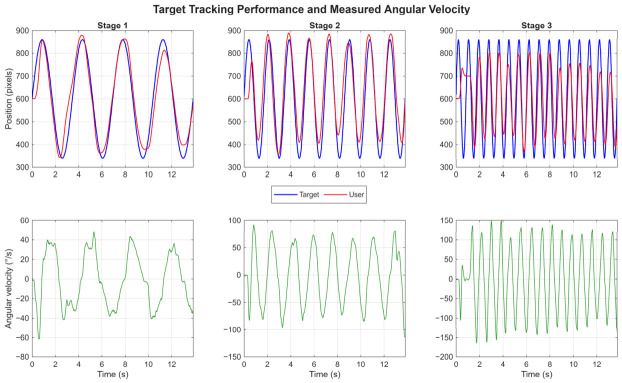
Target tracking performance and corresponding angular velocity across three motion stages of the gamification protocol (S1, coplanar, Env A). (**Top row**): Screen position of the visual target (blue) and subject’s IMU-controlled cursor (red), showing increasing oscillation frequency from Stage 1 (0.2 Hz) through Stage 2 (0.4 Hz) to Stage 3 (0.8 Hz). (**Bottom row**): Measured angular velocity from the gyroscope, demonstrating proportionally increasing peak velocities required to track the faster-moving targets (leading to greater motion artifacts induced in both the primary and secondary signal).

Corresponding data from the (apparatus mounted) IMU is shown in Figure 11, which shows the target tracking performance and corresponding angular velocity across three motion stages of the gamification protocol (0.2 Hz, 0.4 Hz, 0.8 Hz). Aside from generally adequate tracking performance (tracking was more accurate in frequency than in amplitude during Stage 3), proportionally increasing peak angular velocities required to track the faster-moving targets can be noticed. Those, subsequently, lead to greater motion artifacts induced in the primary signal.

**Figure 12:**
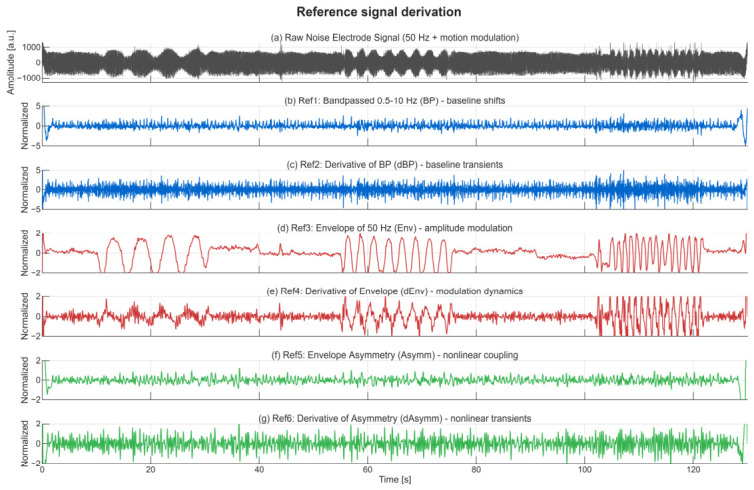
Complete set of reference signals derived from a floating pair of noise electrodes (S1, coplanar, Env A). (**a**) Raw signal. (**b**,**c**) Band-pass-derived references. (**d**,**e**) Envelope-derived references. (**f**,**g**) Asymmetry-derived references. All reference signals are normalized and zero-meaned for input to the NLMS adaptive filter.

Since the raw secondary signal is not suitable for usage as an NLMS reference signal due to high 50 Hz content, reference signals must be extracted from it. A total of six reference signals are extracted, as shown in Figure 12. In contrast to the illustrative Figure 4 (S2, coplanar configuration, Env A), dominance of envelope-based references is clear, with asymmetry-based references showing minimal amplitude. This variability across subjects even in the same environment and configuration (session setup) underscores the value of the multi-reference approach, as the relative contribution of each reference type depends on individual electrode–skin coupling characteristics, as well as specific movement patterns.

**Figure 13:**
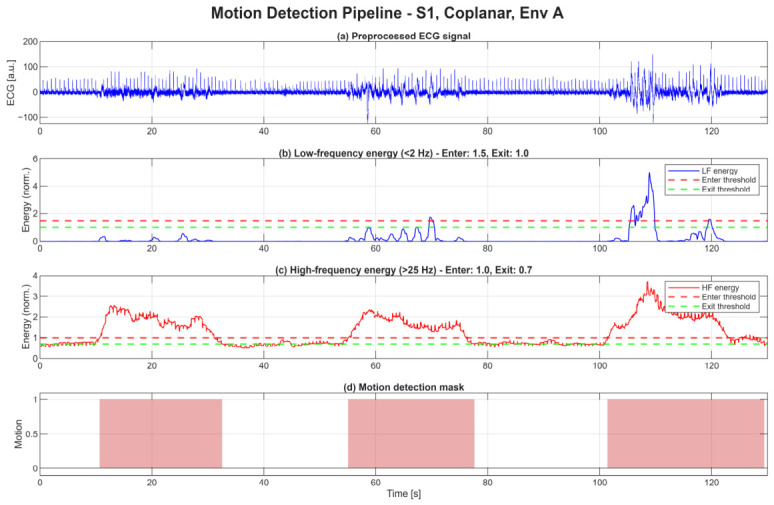
Automatic motion detection pipeline for selective adaptive filter activation (S1, coplanar, Env A). (**a**) Preprocessed cECG signal with visible motion artifacts during movement periods. (**b**) Normalized low-frequency (LF) energy (<2 Hz) computed over sliding windows, capturing baseline wander and slow drift components characteristic of motion; hysteresis thresholds (enter: 1.5, exit: 1.0) prevent rapid state switching. (**c**) Normalized high-frequency energy (>25 Hz) tracking broadband noise increases during movement, with separate threshold levels (enter: 1.0, exit: 0.7). (**d**) Combined binary motion detection mask derived from either energy metric exceeding its threshold.

Since the cECG signal (acquired through primary electrodes) in the resting periods is already of adequate quality, automatic motion detection is performed to enable selective application of the adaptive filter only during artifact-corrupted segments. This enables preserving signal integrity during rest periods and avoids overtraining the algorithm. Noise references are not used to estimate motion segments since, as established earlier, interference in the secondary signal constitutes a superset of the interferences present in the primary signal. Consequently, the noise channel may exhibit significant modulation from sources (particularly 50 Hz mains coupling) that minimally affect the cECG, resulting in false-positive motion detection and unnecessary adaptive filtering. The detection algorithm employs a dual-band energy approach, computing normalized energy in both low-frequency (<2 Hz) and high-frequency (>25 Hz) bands over sliding windows, shown in Figure 13. Low-frequency energy captures baseline wander and slow drift characteristics of motion artifacts, while high-frequency energy detects broadband noise and increases during movement. Hysteresis thresholds are applied to each band independently (low-frequency: enter 1.5, exit 1.0; high-frequency: enter 1.0, exit 0.7) to prevent rapid state oscillation near threshold boundaries. These thresholds have been set empirically. The final motion mask is generated by logical OR of both detectors, flagging a segment as motion-affected if either energy metric exceeds its threshold. This dual-band strategy ensures robust detection across the range of motion frequencies tested: slow movements predominantly elevate low-frequency energy, while fast movements primarily affect the high-frequency band.

**Figure 14:**
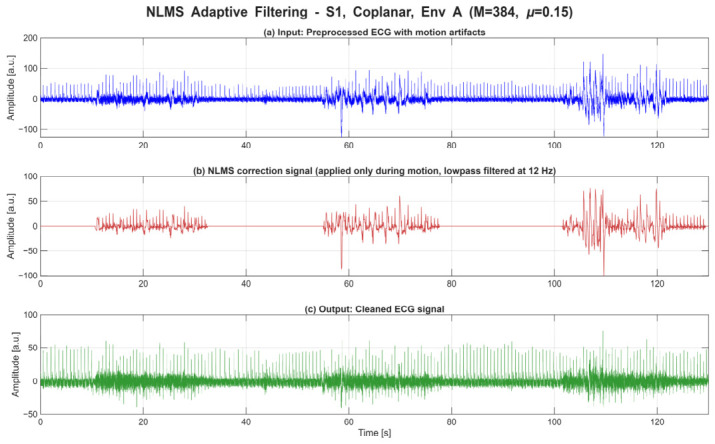
NLMS adaptive filtering results for motion artifact removal (S1, coplanar, Env A; filter order *M* = 384, step size *μ* = 0.15). (**a**) Preprocessed cECG showing motion artifacts during controlled movement periods. (**b**) Correction signal generated by the multi-reference NLMS filter, selectively applied only during detected motion segments and low-pass filtered at 12 Hz to preserve ECG frequency content (particularly R peaks); the filter adapts its output magnitude to match artifact severity across different motion frequencies. (**c**) Cleaned ECG output after subtraction of the correction signal, demonstrating substantial artifact reduction while maintaining QRS complex morphology.

Figure 14 presents the NLMS adaptive filtering process results. The correction signal generated by the multi-reference NLMS filter (*M* = 384, *μ* = 0.15) is applied selectively during detected motion segments and low-pass filtered at 12 Hz to reduce attenuation of QRS complexes. The filter adapts its output to match the varying artifact characteristics across motion conditions, producing larger corrections during the more severely corrupted fast motion segments.

**Figure 15:**
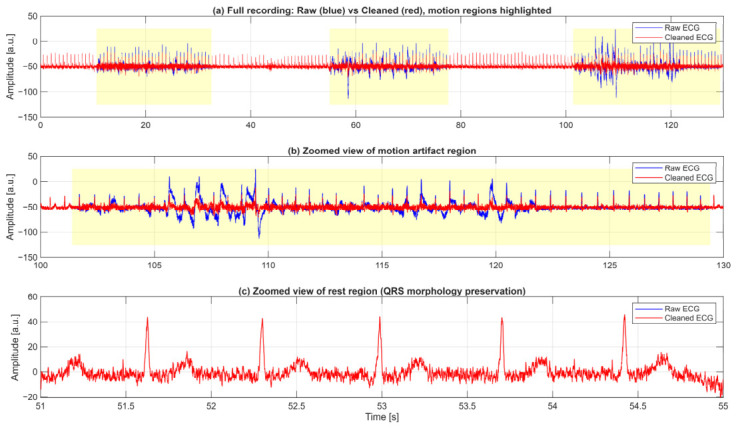
Before/after comparison of adaptive filtering performance (S1, coplanar, Env A). (**a**) Full recording showing raw cECG (blue) overlaid with the cleaned signal (red); yellow shading indicates motion artifact regions across three movement stages, with an overall RMS reduction of 57.2%. (**b**) Zoomed view of the high-frequency motion segment (100–130 s), demonstrating effective artifact suppression even during rapid oscillations, while preserving visible QRS complexes. (**c**) Zoomed view of a rest segment (51–55 s), confirming that adaptive filtering preserves ECG morphology when no correction is required. The raw and cleaned signals remain virtually identical, indicating no signal distortion during stationary periods.

The effectiveness of the adaptive filtering approach is further illustrated in Figure 15, which directly compares raw and cleaned signals. An overall root mean square (RMS) reduction of 57.2% is achieved during motion periods, with visible artifact suppression across all three movement stages. RMS is computed as:

(8) $$\mathrm{RMS}\left(x\right)=\sqrt{\frac{1}{N}\sum_{n=1}^{N}x^{2}\left[n\right]}$$

The corresponding Δ*RMS*% metric is computed as:

(9) $$\Delta RMS\%=\frac{RMS_{raw}-RMS_{filt}}{RMS_{raw}}*100$$

The zoomed view of the high-frequency motion segment (100–130 s) demonstrates that the algorithm successfully reduces artifact amplitudes while preserving identifiable QRS complexes, even during the most challenging rapid oscillations. Notably, the zoomed rest segment (51–55 s) confirms that signal morphology remains unaltered when no motion is detected. The raw and cleaned traces are virtually indistinguishable. This ensures that the adaptive filter improves signal quality during motion without introducing distortion during stationary periods.

**Figure 16:**
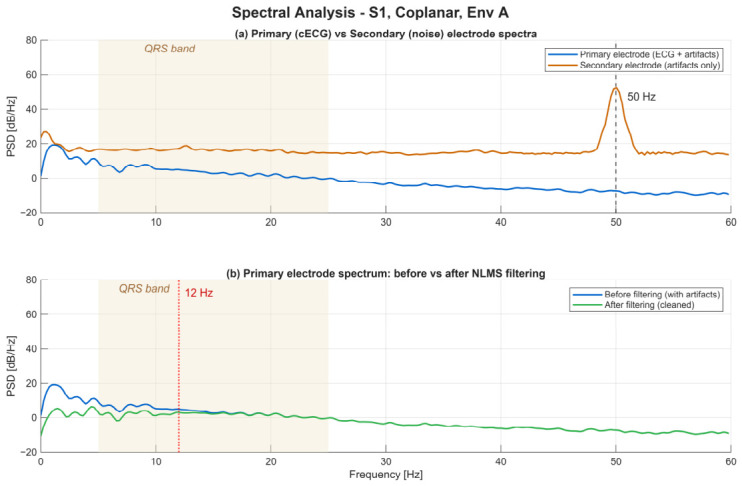
Spectral analysis of primary and secondary signals (S1, coplanar, Env A). (**a**) Comparison of power spectral density between primary (cECG) and secondary (noise) electrodes. (**b**) Primary electrode spectrum before and after NLMS adaptive filtering.

Spectral analysis confirms the distinct characteristics of the primary and secondary signals (Figure 16). The secondary (noise) signal exhibits substantially higher power across most frequencies, with a prominent 50 Hz peak from mains coupling that is absent in the primary signal. After NLMS adaptive filtering, the primary signal spectrum shows selective attenuation below the 12 Hz correction cutoff frequency, where motion artifacts predominantly reside, while power within the QRS band remains mostly preserved. This frequency-selective artifact reduction validates the low-pass filtering strategy applied to the correction signal, ensuring that the adaptive filter targets motion-induced disturbances without compromising the spectral components essential for R-peak detection.

While RMS reduction in interference is significant, even more important in the case of biopotentials, such as cECG, is the ability to reliably extract physiological information. The *F1* score for R-peak detection, computed against manually verified peaks from the gel surface-contact electrode reference, serves as the primary performance metric, as accurate R–R interval measurement is fundamental to HRV analysis and arrhythmia detection. R-peak matching tolerance is set to 50 ms. Detection performance is quantified using sensitivity (*Se*), positive predictive value (*PPV*), and the *F1* score. Sensitivity and *PPV* are defined as:

(10) $$Se=\frac{TP}{TP+FN},PPV=\frac{TP}{TP+FP}$$

where *TP*, *FP*, and *FN* denote true positives, false positives, and false negatives, respectively. The *F1* score is computed as the harmonic mean of *Se* and *PPV*:

(11) $$F_{1}=\frac{2\cdot TP}{2\cdot TP+FP+FN}=\frac{2\cdot Se\cdot PPV}{Se+PPV}$$

While Table 3 reports the *F1* score as the primary metric, the complete set of performance metrics, including *Se* and *PPV*, is provided in Appendix C.

**Figure 17:**
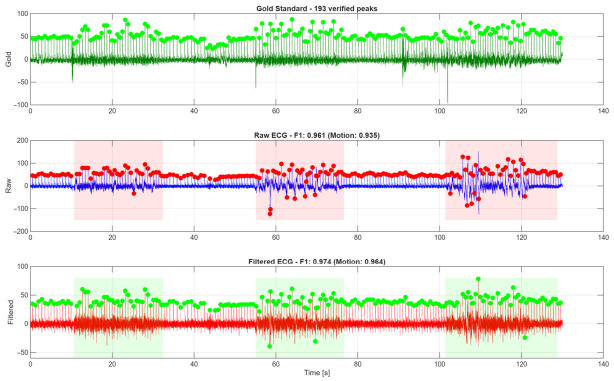
R-peak detection performance comparison between raw and adaptively filtered cECG (S1, coplanar, Env A). (**Top**): Gold standard reference from gel surface-contact electrodes with 193 manually verified R-peaks. (**Middle**): Raw cECG with detected peaks using Pan–Tompkins [50] algorithm (red markers) showing an overall *F1* score of 0.961, degrading to 0.935 during motion segments (red shading). (**Bottom**): NLMS filtered cECG with detected peaks using Pan–Tompkins algorithm (green markers) demonstrating improved detection with overall *F1* score of 0.974 and motion-segment (green shading) *F1* of 0.964. The adaptive filtering notably improves detection reliability during motion.

**Figure 18:**
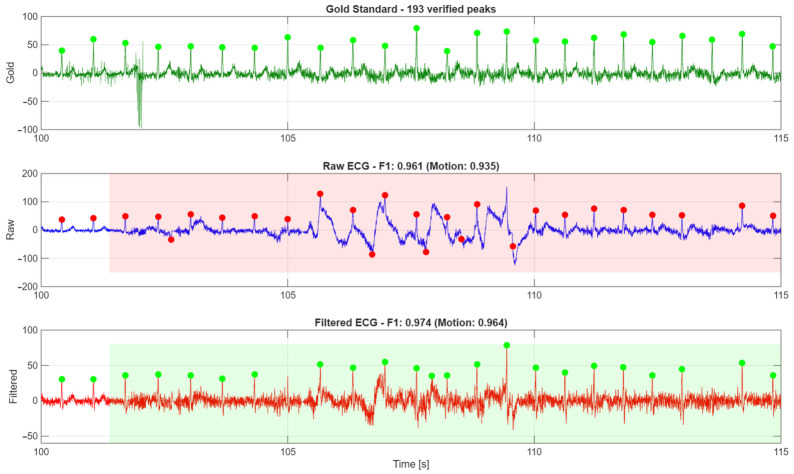
Zoomed-in view (100–115 s) of the high-frequency motion segment from Figure 17 to better illustrate peak alignment (filtered and raw compared to gold standard) and detection errors during motion.

Figure 17 shows detected R peaks over the entire range of the measurement, with a total of 193 manually verified peaks on the gold standard reference signal (top). Raw primary cECG signal contains significant motion artifacts, which negatively impact the detection, as shown in the middle plot. Ground-truth R-peak locations were established by applying the Pan–Tompkins [50] algorithm to the gel surface-contact reference signal, followed by manual verification and correction by the first author. The gel electrode signal was reviewed in sequential 5–10 s segments, with each algorithmically detected peak visually inspected against the raw waveform. Mislocated peaks were repositioned to the true R-peak apex, false detections were removed, and missed peaks were added manually. R-peak detection on both the raw and filtered cECG signals was performed automatically using the Pan–Tompkins algorithm without manual intervention, ensuring unbiased comparison between conditions. The bottom plot showcases NLMS-filtered cECG with detected peaks using the Pan–Tompkins algorithm, demonstrating improved detection with overall *F1* score of 0.974 and motion-segment *F1* of 0.964, alongside 57.2% RMS reduction. To better illustrate the differences between the two signals (raw and cleaned), Figure 18 presents a zoomed-in view of the high-frequency motion segment from Figure 17. Examination of the zoomed segment reveals a subtle but clinically relevant improvement: in the raw cECG, detected peaks are occasionally displaced from their true locations due to artifact interference, appearing near but not precisely at the actual R-peak positions. The cleaned signal corrects most of these misalignments, improving the alignment of detections with the QRS complexes. This improvement may not be fully captured by the *F1* metric (which tolerates small temporal deviations within the matching window), yet is readily apparent upon visual inspection and would be immediately recognized by a trained clinician as improved signal quality.

### 3.2. Full Results for All Subjects and Sessions

#### 3.2.1. Data Curation and Exclusions

Of the 28 planned recording sessions (seven subjects × four conditions), four were excluded due to technical issues unrelated to the algorithm: battery voltage degradation caused irregular sample timing in four sessions (S5, stacked, and S7, Env B sessions). In two measurement sessions (S5, coplanar, Env A; S6, coplanar, Env A) gel electrode signal degraded due to motion during Stage 3, hence these recordings were truncated to the valid portion prior to signal loss. One subject (S6) exhibited suboptimal electrode contact, occasionally lifting their hand from the sensor during the gamification protocol. These sessions were retained in the analysis to evaluate algorithm behavior under challenging real-world conditions, where consistent electrode contact cannot be guaranteed.

#### 3.2.2. Performance Evaluation

Table 3 summarizes the adaptive filtering performance across all seven subjects and four experimental conditions.

**Figure 19:**
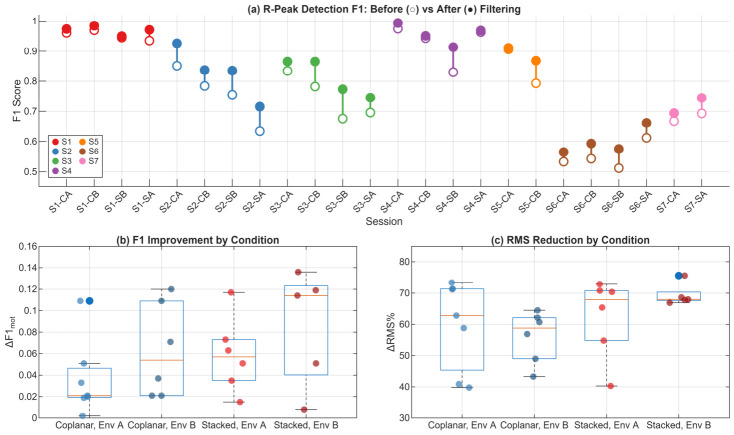
Summary of filtering performance across all recording sessions. (**a**) *F1* score before (open circles) and after (filled circles) adaptive filtering, with sessions grouped by subject (colors) and ordered by subject–configuration–environment. All 24 sessions showed improvement. (**b**) *F1* score improvement (Δ*F1*_mot_) by experimental condition. (**c**) RMS amplitude reduction (Δ*RMS*%) by experimental condition. Box plots show median and interquartile range; individual sessions are overlaid as points.

The adaptive filtering algorithm improved R-peak detection across all 24 recording sessions (Figure 19a). In motion artifact regions, the *F1* score improved by Δ*F1*_mot_ = +0.059 ± 0.043 (median: +0.051, 95% confidence interval CI: [0.043, 0.076]), with improvements ranging from +0.002 to +0.136. RMS amplitude in motion regions was reduced by 61.5 ± 11.3% (median: 65.0%, 95% CI: [57.0%, 65.6%]), with improvements ranging from 39.8% to 75.6%. The reported 95% confidence intervals were computed using a normal approximation across the 24 sessions. As sessions within the same subject are not fully independent (up to four sessions per subject), these intervals should be interpreted as descriptive summaries of the observed variation rather than formal inferential bounds. Given that the limited and unbalanced sample (seven subjects with two to four sessions each) precludes robust formal inferential analysis, we report descriptive statistics and emphasize the consistency of improvement across all subjects and sessions. Notably, all 24 sessions exhibited positive improvement in both metrics, with 19 of 24 sessions (79%) achieving a greater than 50% RMS reduction. No session showed signal degradation, confirming the algorithm’s “do no harm” characteristic.

To complement the session-level descriptive analysis with subject-level inference, paired non-parametric tests were applied to the per-subject median improvement across sessions. Aggregating to one value per subject (median Δ*F1*_mot_ per subject) yielded a sign test result of *p* = 0.016 (all seven subjects were positive; exact binomial, one-tailed) and a Wilcoxon signed-rank test result of *p* = 0.016 (W = 28, one-tailed), indicating that the improvement in R-peak detection *F1* score was consistent across all subjects. The same procedure applied to ΔRMS% yielded identical results (sign test *p* = 0.016, Wilcoxon *p* = 0.016, W = 28), confirming that artifact energy reduction was unanimously positive across all subjects. These results should be interpreted with the following caveats:

1. With n = 7, the minimum attainable one-tailed *p*-value is 0.016, so the result primarily reflects the unanimous direction of effect rather than providing strong inferential evidence about the population;
2. The per-subject aggregation discards within-subject session-level variation captured by the session-level analysis;
3. The unbalanced repeated-measures structure (2–4 sessions per subject) means sessions contributing to each subject’s median are not equally informative;
4. These tests are therefore provided as a formal complement to the descriptive session-level analysis, not as a replacement; population-level inference would require a substantially larger and balanced cohort.

To examine whether improvement magnitude depended on baseline signal quality, Spearman’s rank correlation was computed between baseline *F1* score and improvement metrics. Including all 24 sessions, the correlation was moderate but not statistically significant (*p* < 0.05) for Δ*F1*_mot_ (*r*_s_ = −0.33, *p* = 0.113), though significant for Δ*RMS*% (*r*_s_ = −0.56, *p* = 0.005). Two outlier conditions were identified: one session (S5, coplanar, Env A) where the gel reference electrode degraded during recording, and one subject (S6) who exhibited intermittent electrode lift-off during the protocol. Excluding these outliers (*n* = 19), significant negative correlations emerged for both Δ*F1*_mot_ (*r*_s_ = −0.59, *p* = 0.007) and Δ*RMS*% (*r*_s_ = −0.49, *p* = 0.035), indicating that the algorithm provided greater improvement for more severely degraded signals.

Figure 19b,c shows the distribution of improvement metrics across experimental conditions. While stacked configurations showed numerically higher median Δ*F1*_mot_ (stacked, Env A: 0.073; stacked, Env B: 0.114) compared to coplanar configurations (coplanar, Env A: 0.033; coplanar, Env B: 0.053), substantial within-group variability precludes definitive conclusions about configuration effects, other than the effect of increased PLI for larger electrode loop areas. RMS reduction was notably consistent across all conditions, with median values ranging from 58% to 69%, indicating robust algorithm performance independent of electrode geometry or electromagnetic environment.

Full tables of results are included in Appendix C.

## 4. Discussion

This study presented a double electrode pair approach for motion artifact removal in non-contact ECG systems, utilizing a pair of secondary (noise) electrodes to derive reference signals for adaptive filtering. The method was validated across seven subjects, two electrode configurations, two environments with different mains interference levels, and three stages of motion frequency spanning slow to fast movements.

As shown by the results, slower movements (0.2 Hz) produced artifacts of lower amplitude in the primary electrodes, resulting in reduced need for correction. Faster movements (0.4 Hz, and especially 0.8 Hz) generated substantial artifacts that were effectively captured by the reference signals. Application of the multi-reference NLMS adaptive filter yielded consistent improvements in both signal quality and R-peak detection performance. RMS amplitude in motion regions was reduced by 61.5 ± 11.3% (range: 39.8–75.6%), while *F1* scores improved by +0.059 ± 0.043 in motion regions (range: +0.002 to +0.136). All 24 sessions showed positive improvement in both metrics. These improvements translate directly to more reliable heart rate and HRV measurements in the presence of motion. HRV analysis requires accurate R–R interval measurement, as missed or spuriously detected beats corrupt HRV metrics disproportionately. To further evaluate the temporal precision of R-peak localization, additional analyses were performed. When the matching tolerance was reduced from 50 ms to 20 ms, raw signal *F1* often decreased substantially (e.g., from 0.918 to 0.850 in S1, stacked, Env B), indicating that many detected peaks were temporally displaced from the true R-peak location due to artifact-induced waveform distortion. In contrast, filtered signal *F1* showed only a modest decrease (0.926 to 0.906 under the same conditions), confirming that artifact removal improved not only detection sensitivity but also temporal precision.

The algorithm demonstrated consistent performance across both electrode configurations and electromagnetic environments. While stacked configurations showed numerically higher median Δ*F1*_mot_ (0.071) compared to coplanar (0.049), and Environment B showed slightly greater improvement than Environment A, these differences were substantially smaller than the inter-subject variability. RMS reduction was similar across all conditions (coplanar: 58.1 ± 10.6%; stacked: 65.6 ± 9.9%; Env A: 60.7 ± 12.0%; Env B: 62.1 ± 8.1%), with overlapping distributions indicating no meaningful configuration or environment dependence. The dominant source of variation was individual subject differences (reflecting factors such as skin properties, electrode contact quality, and motion execution) rather than experimental conditions. This robustness is a practical advantage: the algorithm generalizes across electrode geometries and electromagnetic environments without requiring parameter tuning, simplifying deployment in real-world wearable applications where these factors cannot be tightly controlled.

A significant negative correlation was observed between baseline signal quality and improvement magnitude. For the clean dataset (n = 19, excluding sessions with gel electrode failure and occasional electrode lift-off), Spearman’s correlation yielded *r*_s_ = −0.59 (*p* = 0.007) for Δ*F1*_mot_ and *r*_s_ = −0.49 (*p* = 0.035) for Δ*RMS*%. This indicates that the algorithm provides proportionally greater benefit for more severely degraded signals while preserving already-clean recordings, which is a desirable adaptive characteristic. Sessions with high baseline *F1* (>0.95) showed minimal absolute improvement due to ceiling effects, yet still achieved 40–60% RMS reduction without introducing distortion. Conversely, sessions with poor baseline quality (*F1* < 0.70) improved by up to +0.136 in *F1* score. The one exception was Subject 6, who exhibited intermittent electrode lift-off during the protocol; despite low baseline *F1* (0.51–0.61), improvement was modest (Δ*F1*_mot_: 0.02–0.05), confirming that the algorithm effectively targets motion-induced capacitive coupling artifacts but cannot compensate for complete signal loss due to electrode lift-off. This distinction between correctable artifacts (motion modulation) and uncorrectable failures (electrode lift-off) has practical implications for wearable system design, where electrodes’ mechanical properties and retention become the limiting factor rather than signal processing.

The key advantage of the proposed method is its independence from the physical source of the motion artifact. IMU-based adaptive filtering approaches can only detect motion of the sensor or subject itself, remaining largely insensitive to environmental disturbances that perturb the surrounding electric field [36,37]. More recently, ETI-based methods have been proposed to capture interface variations associated with motion; however, ETI exhibits the highest correlation with locally induced electrode artifacts and substantially weaker correlation with global body motion or external electric-field perturbations [18,36,38,39]. In non-contact ECG applications, artifacts arise not only from subject movement but also from movement of nearby persons, conductive objects, or changes in the ambient electromagnetic environment scenarios common in automotive applications with passenger movement, or clinical and home monitoring settings where healthcare workers, medical equipment or family members may disturb the measurement. The noise electrode approach captures the electromagnetic consequence of any such disturbance, regardless of its origin. Preliminary measurements with artifacts from external sources (nearby movement of people and equipment) showed the algorithm maintained signal quality without degradation, though improvement was marginal due to the smaller artifact amplitudes. This is consistent with algorithm effectiveness observed at 0.2 Hz controlled motion, where induced motion artifacts are minimal. Systematic quantitative validation of the method’s performance against environmentally induced artifacts, with controlled and reproducible external disturbance sources, remains a direction for future work. It is also worth noting that in two sessions, the gel surface-contact reference electrodes degraded under the same motion conditions that the non-contact system tolerated, requiring truncation of those recordings. This underscores that non-contact electrodes, despite their weaker coupling, can exhibit greater mechanical robustness to motion than conventional gel electrodes in certain scenarios.

Another advantage of the proposed method is that the complete processing pipeline is appropriate for real-time implementation. All filtering operations can be implemented causally using infinite impulse response (IIR) filters with acceptable phase delay. The NLMS algorithm is inherently sample by sample, requiring only O(M) multiply–accumulate (MAC) operations per sample, where O() denotes asymptotic computational complexity. For the parameters used in this study (*M* = 384, six references), the computational load is approximately 4600 multiply–accumulate operations per sample. This is well within the capability of modern embedded processors, including the Cortex-M4 class microcontrollers commonly used in wearable devices. This is an advantage over computationally intensive offline methods, such as EMD-, EEMD-, and BSS-based approaches.

The use of six complementary reference signals, each capturing different aspects of motion-induced interference, proved essential for robust performance across varied conditions. Analysis revealed that no single reference dominated across all subjects, configurations, and motion frequencies. This validates the multi-reference design philosophy, wherein the adaptive filter automatically weights each reference according to its instantaneous contribution.

The envelope asymmetry reference, derived from the difference between positive and negative half-cycle envelopes of the 50 Hz interference, represents a novel contribution to cECG signal processing. The physical basis for this reference lies in the non-linear nature of capacitive coupling: positive and negative voltage excursions create different electric-field distributions and may be affected differently by motion-induced geometry changes. In several sessions, particularly those involving fast or abrupt motion, the asymmetry reference modeled ECG artifacts better than the symmetric envelope, confirming that it captures complementary information. To our knowledge, this reference type has not been previously described in the context of motion artifact removal.

The proposed approach requires only an additional electrode pair and matched AFE circuitry, in contrast to [41] where a novel designed electrode structure is required. The noise electrodes can be integrated into the same substrate as the signal electrodes, adding minimal size, weight, and cost to the overall system. The matched AFE design ensures that any frequency-dependent effects are common to both channels, simplifying the adaptive filtering task. Similar adaptive noise cancelation approaches using adjacent non-contact electrodes have been proposed [42,43], where reference signals are derived from the difference between neighboring cECG sensor pairs. However, these methods suffer from residual cECG content in the reference signal, violating the key assumption for optimal adaptive filtering. A more limited single-reference approach without extensive human subject validation was proposed in [40].

To contextualize the proposed approach within the landscape of existing methods, Table 4 provides a structured qualitative comparison of the main reference-assisted motion artifact removal strategies for non-contact ECG. The table contrasts the approaches across hardware requirements, causal construction, computational complexity, cardiac content in the reference signal, and sensitivity to different artifact mechanisms. Because a matched quantitative comparison would require hardware not present in the current acquisition setup (body-mounted IMU sensors for an IMU-referenced baseline, and dedicated impedance injection and measurement circuitry for an ETI-referenced baseline), the table is structured around architectural and mechanistic properties that are well established in the literature [18,36,37,38,39,40,41,42,43] rather than empirical performance figures. Such direct benchmarking is identified as a priority for future work.

One of the limitations of this research is dependence on mains interference for envelope-type references. Specifically, the envelope and asymmetry references (Env, dEnv, Asymm, dAsymm) derive their information from amplitude modulation of the mains coupling and would provide diminished contribution in environments with very low 50 Hz field strength. The baseline-type references (BP, dBP), however, capture direct motion-induced signal variations independent of mains coupling and would remain fully functional. The multi-reference NLMS architecture inherently accommodates this scenario: the adaptive filter assigns near-zero weights to uninformative references, allowing the remaining references to drive artifact estimation. The method is therefore expected to degrade gracefully rather than fail entirely, albeit with reduced artifact suppression capability. In such cases, in spite of application-specific dependence of overall performance on the extent to which motion artifacts manifest as baseline-type disturbances, the algorithm would require no additional parameter tuning. Testing across two environments with different mains interference levels showed consistent performance, suggesting robustness within typical indoor settings. However, extremely low-interference environments were not evaluated. Furthermore, all measurements were performed using a standardized wooden rotation apparatus with controlled motion frequencies. While this approach enabled systematic evaluation and reproducible comparisons, it does not fully represent real-world usage scenarios, such as driving, sleeping, or daily activities. The gamification protocol ensured consistent motion patterns across subjects but may not capture the full diversity of motion artifacts encountered in practice. The cohort of seven subjects is appropriate for a proof-of-concept validation under the tightly controlled conditions described above; however, the sample size limits population-level generalization, and larger-cohort validation with more diverse populations and natural motion scenarios is an explicit priority for the next stage of this line of work.

Another limitation concerns the fact that the NLMS algorithm involves several parameters that were selected empirically based on preliminary testing. While the chosen parameters performed well across all sessions in this study, they may require adjustment for different hardware configurations, electrode geometries, or application scenarios. The robustness of the method to parameter variations, and the development of automatic parameter tuning strategies remain topics for future investigation. Although a comprehensive sensitivity analysis was not performed, preliminary parameter exploration was conducted during algorithm development. The filter order M was tested from 32 to 384 samples; performance remained comparable down to approximately 64 samples, with only marginal degradation at lower orders, suggesting feasibility for resource-constrained embedded implementations. The step size μ produced similar results across the range 0.1–1.0, higher values yielded even higher RMS reductions (up to 5% higher), but with minimal effect on Δ*F1*_mot_ (+0.01 to +0.05) and increased risk of filter instability. The conservative value of μ = 0.15 was selected to prioritize stable convergence across diverse recording conditions at the cost of marginally lower artifact suppression. The correction low-pass filter cutoff was evaluated between 10 and 15 Hz, with 12 Hz representing a balanced trade-off between artifact suppression and QRS preservation; performance was acceptable throughout this range. The regularization parameter ε had no observable effect across the tested range (10^−1^ to 10^−4^). The partial correction factor α performed best in the range 0.88–0.95, and the leakage factor γ in the range 10^−2^ to 10^−3^. Overall, algorithm performance was not brittle around the selected parameter values, which supports the method’s practical applicability across different hardware configurations.

One parameter in particular warrants separate discussion, as it represents a more fundamental trade-off than the others. The correction signal low-pass filter, implemented to preserve QRS morphology, necessarily limits the removal of high-frequency artifact components. Motion artifacts with significant energy above the filter cutoff frequency (12 Hz) will be incompletely removed. This trade-off between artifact removal and QRS preservation is inherent to the approach and may require application-specific tuning.

For applications where continuous monitoring through periods of activity is required, like stress monitoring, sleep staging with movement, and automotive driver monitoring, the proposed method offers a path to more reliable measurements without constraining user behavior or requiring specialized motion sensors.

Several directions for future investigation emerge from this work:

1. Validation in real-world application settings (such as driving, sleep monitoring, ambulatory use), as well as a comprehensive sensitivity analysis would establish practical utility.
2. Systematic quantitative validation of the method’s performance against environmentally induced artifacts, with controlled and reproducible external disturbance sources, would confirm the source-agnostic property of the method.
3. Comparative quantitative benchmarking against existing reference-assisted artifact removal methods (such as IMU- and ETI-based adaptive filtering), under matched motion protocols and hardware configurations, would quantify the method performance within the broader landscape of cECG artifact suppression techniques. Implementing such a comparison would require body- or electrode-mounted inertial sensors for an IMU-referenced baseline, and dedicated impedance injection and measurement circuitry for an ETI-referenced baseline; both constitute separate experimental campaigns that are deferred to follow-on work.
4. Embedded implementation with power and latency characterization would confirm deployment feasibility.
5. Automatic parameter adaptation based on signal characteristics could improve robustness across diverse conditions.
6. Extension to other biopotentials measured with non-contact electrodes (such as EMG) may broaden applicability.

## 5. Conclusions

This study presented a proof-of-concept validation of a novel approach to motion artifact removal in non-contact ECG systems, using a double pair of electrodes where dedicated floating noise electrodes provide reference signals for adaptive filtering. Six reference signals were derived from the noise electrode channel, capturing baseline shifts, envelope modulation, and envelope asymmetry of the mains interference, and processed through a multi-reference NLMS adaptive filter.

The method was validated across seven subjects, two electrode configurations, two electromagnetic environments, and three motion frequencies (movement stages). Across all 24 sessions, the algorithm consistently improved R-peak detection *F1* score by +0.059 ± 0.043 in motion regions while reducing RMS artifact amplitude by 61.5 ± 11.3%, with no session showing degradation.

Three key advantages distinguish this approach from existing methods. First, artifact removal is designed to be agnostic to the source of interference as the noise electrodes capture the electromagnetic consequence of any disturbance regardless of its origin, though the present study validated this primarily for subject-induced motion. Second, the processing pipeline is fully compatible with real-time embedded implementation, requiring no computationally intensive offline processing. Third, the approach requires only an additional pair of electrodes with matched AFE circuitry, avoiding the need for supplementary motion sensors.

The envelope asymmetry reference, exploiting non-linear capacitive coupling effects, represents a novel contribution that proved particularly effective for non-linear motion artifacts. The multi-reference strategy ensured robust performance across diverse conditions without requiring manual reference selection.

Limitations include dependence on mains interference for envelope-type references, validation only in controlled laboratory conditions, and empirically selected algorithm parameters. Future work will address real-world validation, competitive benchmarking, embedded implementation, and extension to other biopotentials measured with non-contact electrodes.

The proposed method offers a practical path toward reliable non-contact ECG monitoring in the presence of motion, supporting applications in automotive, wearable, and unobtrusive health monitoring where artifact-free measurement cannot be guaranteed.

## Figures and Tables

**Table 1:** NLMS algorithm parameters used in this study.

	Symbol	Value	Description
Filter order	*M*	384	Samples of reference history per reference
Step size	*μ*	0.15	NLMS adaptation rate
Regularization	*ε*	0.1	Prevents division by zero
Leakage factor	*γ*	0.001	Weight decay rate
Partial correction factor	*α*	0.9	Correction scaling factor
Correction low-pass corner frequency	*f*_c_	12 Hz	QRS preservation cutoff

**Table 2:** Demographic characteristics of the subjects, including age, sex (F—female, M—male), and body mass index (BMI).

	Age	Sex	BMI [kg/m^2^]
Subject 1	30	M	23.9
Subject 2	67	M	31.2
Subject 3	22	F	20.0
Subject 4	58	F	23.2
Subject 5	56	F	24.0
Subject 6	56	M	28.7
Subject 7	21	F	22.7

**Table 3:** Adaptive filtering performance across all subjects and sessions. Δ*F1*_mot_ indicates the change in R-peak detection *F1* score during motion segments (filtered minus raw), with positive values representing improved detection accuracy. ∆*RMS*% denotes the percentage reduction in root mean square amplitude during motion periods, quantifying artifact suppression effectiveness. Results are presented for coplanar and stacked electrode configurations tested in two environments with differing 50 Hz interference levels (Environment A: high interference; Environment B: low interference). Hyphen (-) indicates sessions excluded due to data acquisition issues: S5 and S7 experienced battery-related timing inconsistencies causing irregular sample intervals. Asterisk (*) denotes truncated measurements due to gel electrode degradation during motion stage 3.

	Coplanar, Env A		Coplanar, Env B		Stacked, Env A		Stacked, Env B	
	∆*F1*_mot_	∆*RMS*%	∆*F1*_mot_	∆*RMS*%	∆*F1*_mot_	∆*RMS*%	∆*F1*_mot_	∆*RMS*%
Subject 1	0.021	58.8	0.021	56.9	0.051	65.4	0.008	68.6
Subject 2	0.109	71.4	0.071	62.1	0.117	70.4	0.114	67.8
Subject 3	0.051	62.8	0.120	60.7	0.073	70.8	0.136	66.9
Subject 4	0.033	40.9	0.021	64.5	0.015	40.3	0.119	68.0
Subject 5	0.002 *	39.8	0.109	43.3	-	-	-	-
Subject 6	0.019 *	73.4	0.037	49.0	0.035	72.9	0.051	75.6
Subject 7	0.020	71.2	-	-	0.063	54.8	-	-

**Table 4:** Qualitative comparison of reference-assisted motion artifact removal approaches for cECG. ✓ = present/favorable; ✗ = absent/unfavorable; ∼ = partial/conditional; refs = references.

Property	Decomposition-Based (EMD/BSS)	IMU-Referenced Adaptive Filtering	ETI-Referenced Adaptive Filtering	Adjacent-Electrode-Referenced	Proposed Floating Electrode
Additional hardware required	None	IMU on body or electrode	Impedance injection and measurement circuitry	Additional cECG electrode pair	Floating electrode pair + matched AFE
Causal/real-time compatible	✗	✓	✓	Depends on implementation	✓
Computational complexity	High	Low–moderate	Low–moderate	Low	Low
Free from cardiac content in reference signal	N/A (no external reference)	✓	∼ (may contain residual ECG)	✗	✓
Sensitive to subject-induced motion artifacts	✓	✓	∼ (strongest for local electrode motion)	✓	✓
Sensitive to environmentally induced artifacts (external electric-field perturbations)	∼ (depends on decomposition quality)	✗	✗	∼ (residual cECG limits sensitivity)	✓
Dependent on mains (50/60 Hz) for reference generation	✗	✗	✗	✗	∼ (envelope refs require PLI; BP refs do not)

**Figure A1:**
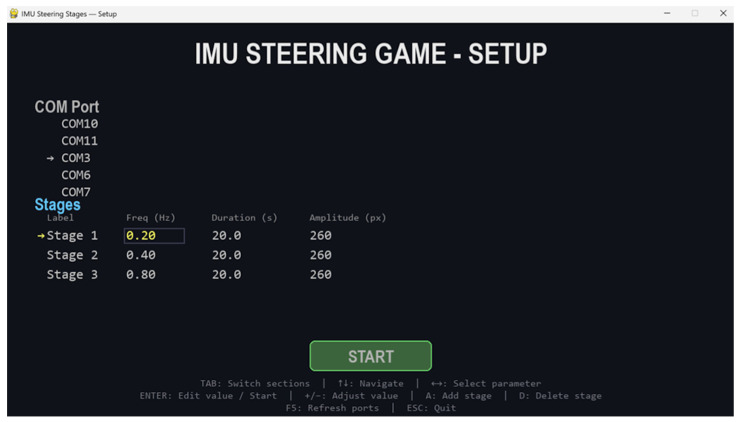
Configuration screen of the gamification interface showing configurable experimental parameters. The interface allows selection of the IMU serial port and definition of motion protocol stages, each specified by oscillation frequency (Hz), duration (s), and target amplitude (in pixels, px).

**Figure A2:**
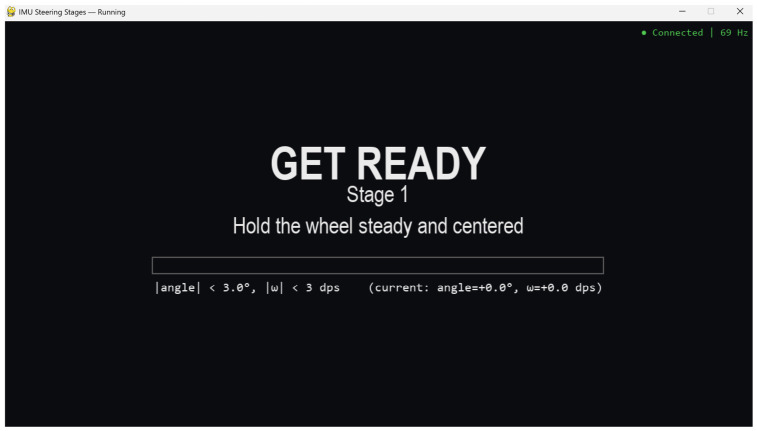
“Get ready” screen of the gamification interface. The progress bar fills as both conditions are continuously satisfied, ensuring consistent initial posture across all stages and subjects. This enforced rest period also provides a clean baseline segment in the recorded data, facilitating reliable segmentation between motion and rest intervals.

**Figure A3:**
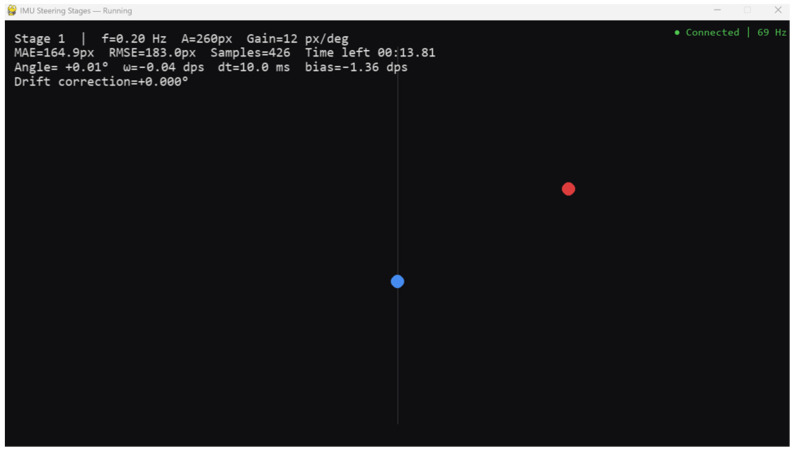
Screenshot of the gamification interface for controlled motion generation. The red circle indicates the target position, moving sinusoidally along the vertical axis, while the blue circle represents the subject’s cursor controlled by steering wheel rotation measured via integrated IMU. Real-time feedback displayed includes current stage parameters (frequency *f* = 0.20 Hz, amplitude *A* = 260 px, *gain* = 12 px/deg, pixels per degree), tracking performance metrics (*MAE* = 164.9 px, *RMSE* = 183.0 px), IMU data (angle, angular velocity ω, sampling interval *dt*, gyroscope bias estimate), and connection status (69 Hz update rate). Subjects are instructed to minimize the distance between target and cursor by rotating the steering wheel, producing standardized, repeatable motion patterns at prescribed frequencies across experimental stages.

## Data Availability

The datasets presented in this article are not readily available due to legal and privacy concerns. Requests to access the datasets should be directed to the corresponding author.

## Abbreviations

The following abbreviations are used in this manuscript:

6-DoF	six degrees of freedom
AAF	anti-aliasing filter
ADC	analog-to-digital converter
AFE	analog front-end
Asymm	envelope asymmetry signal
BP	band-pass signal
BPF	band-pass filter
BSS	blind source separation
cDRL	capacitive driven right leg
cECG	capacitive electrocardiogram
CI	confidence interval
COM	communication (port)
dAsymm	derivative of envelope asymmetry signal
dBP	derivative of band-pass signal
DC	direct current
dEnv	derivative of envelope signal
DSP	digital signal processing
ECG	electrocardiogram, electrocardiography
EEMD	ensemble empirical mode decomposition
EMD	empirical mode decomposition
EMG	electromyogram, electromyography
Env	envelope signal
Env A/B	environment A/B
ETI	electrode–tissue impedance
FN	false negatives
FP	false positives
HF	high frequency
HPF	high-pass filter
HRV	heart rate variability
ICA	independent component analysis
IIR	infinite impulse response
IMU	inertial measurement unit
LF	low frequency
LPF	low-pass filter
MAC	multiply–accumulate
MAE	mean absolute error
NLMS	normalized least mean squares
PCA	principal component analysis
PCB	printed circuit board
PLI	power-line interference
PPV	positive predictive value
PSD	power spectral density
RFI	radio frequency interference
RMS	root mean square
RMSE	root mean square error
S	subject (1, 2, …)
Se	sensitivity
SNR	signal-to-noise ratio
SPP	(Bluetooth) serial port profile
STV	short-time variance
TP	true positives
UART	universal asynchronous receiver-transmitter

## Appendix A. Algorithm Pseudocode

**Reference Signal Generation**	
Input: Secondary electrode signal	**Input:** x_secondary [1..N], *f_s_*
Output: Reference vector	**Output:** x(n) = [x_1_(n); x_2_(n); …; x_6_(n)]
	
Baseline removal	x_noise ← x_secondary − movmedian(x_secondary, *f_s_*)
High-pass filtering	x_noise ← HPF(x_noise, fc = 0.5 Hz)
	
Baseline-type references	
Band-pass filter	x_1_ ← BPF(x_noise, 0.5 Hz, 10 Hz)
Normalize	x_1_ ← x_1_/std(x_1_)
Derivative	x_2_ ← d/dt(x_1_)
Normalize	x_2_ ← x_2_/std(x_2_)
	
Envelope-type references	
Envelope extraction	x_3_ ← LPF(|x_noise|, f_c_ = 4 Hz)
Remove mean, normalize	x_3_ ← (x_3_ − mean(x_3_))/std(x_3_)
Derivative	x_4_ ← d/dt(x_3_)
Normalize	x_4_ ← x_4_/std(x_4_)
	
Asymmetry-type references	
Separate half-cycles	x^+^ ← max(x_noise, 0)
	x^−^ ← |min(x_noise, 0)|
Extract envelopes	env^+^ ← LPF(x^+^, f_c_ = 4 Hz)
	env^−^ ← LPF(x^−^, f_c_ = 4 Hz)
Asymmetry signal	x_5_ ← env^+^ − env^−^
Remove mean, normalize	x_5_ ← (x_5_ − mean(x_5_))/std(x_5_)
Derivative	x_6_ ← d/dt(x_5_)
Normalize	x_6_ ← x_6_/std(x_6_)
**Motion Detection**	
Input: Primary signal	**Input:** d(n), n = 1..N
Output: Motion mask	**Output:** mask(n)
Parameters:	θ_LF_enter, θ_LF_exit, θ_HF_enter, θ_HF_exit
	T_min_motion, T_min_rest
	
Compute LF energy (<2 Hz)	
Low-pass filtering	d_LF ← movmedian(d, *f_s_*/2)
Energy estimation	E_LF ← movvar(d_LF, 1000)
Normalization	E_LF ← E_LF/percentile(E_LF, 95)
	
Compute HF energy (>25 Hz)	
High-pass filtering	d_HF ← HPF(d, f_c_ = 25 Hz)
Energy estimation	E_HF ← movvar(d_HF, 2000)
Normalization	E_HF ← E_HF/percentile(E_HF, 50)
	
Hysteresis–LF channel	
Initialize	inMotion_LF ← false
	**for** n = 1 to N **do**
Exit condition	**if** inMotion_LF and E_LF(n) < θ_LF_exit **then**
	inMotion_LF ← false
Enter condition	**else if** not inMotion_LF and E_LF(n) > θ_LF_enter **then**
	inMotion_LF ← true
	**end if**
Store state	mask_LF(n) ← inMotion_LF
	**end for**
	
Hysteresis–HF channel	
Initialize	inMotion_HF ← false
	**for** n = 1 to N **do**
Exit condition	**if** inMotion_HF and E_HF(n) < θ_HF_exit **then**
	inMotion_HF ← false
Enter condition	**else if** not inMotion_HF and E_HF(n) > θ_HF_enter **then**
	inMotion_HF ← true
	**end if**
Store state	mask_HF(n) ← inMotion_HF
	**end for**
	
Combine channels	mask ← mask_LF or mask_HF
	
Minimum duration enforcement	
Remove short motion	**for** each motion segment **do**
	**if** duration < T_min_motion **then**
	mask(segment) ← false
	**end if**
	**end for**
Fill short gaps	**for** each rest gap **do**
	**if** duration < T_min_rest **then**
	mask(gap) ← true
	**end if**
	**end for**
	
**Multi-Reference NLMS Adaptive Filtering**	
Input: Primary signal, references	Input: d(n), x(n), mask(n), n = 1..N
Output: Artifact estimate	Output: ŷ(n)
Parameters:	M, μ, ε, γ
	
Initialize	w(0) ← 0^6M × 1^
Find motion onsets	onsets ← {n: mask(n−1) = 0 and mask(n) = 1}
	
Main loop	**for** n = M to N **do**
	
Reset at motion onset	**if** n ∈ onsets **then**
	w(n) ← 0^6M × 1^
	**end if**
	
Build reference vector	x(n) ← [x_1_(n:n−M + 1); x_2_(n:n−M + 1); …
(6M × 1)	x_3_(n:n−M + 1); x_4_(n:n−M + 1);
	x_5_(n:n−M + 1); x_6_(n:n−M + 1)]
	
Artifact estimate	ŷ(n) ← wᵀ(n) · x(n)
	
Error for adaptation	e_raw(n) ← d(n) − ŷ(n)
	
NLMS weight update	w(n + 1) ← (1−γ)·w(n) + μ·e_raw(n)·x(n)
(leaky NLMS)	/(|x(n)|^2^ + ε)
	
	**end for**
	
**QRS-Preserving Correction Application**	
Input: Primary signal, estimate, mask	Input: d(n), ŷ(n), mask(n)
Output: Cleaned cECG	Output: e(n)
Parameters:	α, f_c_
	
Scale estimate	ŷ_scaled(n) ← α · ŷ(n)
(overcorrection prevention)	
	
Lowpass filter	ŷ_filt(n) ← LPF(ŷ_scaled, fc)
(QRS preservation)	
	
Hard gating	**for** n = 1 to N **do**
(correction only in motion)	**if** mask(n) = true **then**
	correction(n) ← ŷ_filt(n)
	**else**
	correction(n) ← 0
	**end if**
	**end for**
	
Compute cleaned output	e(n) ← d(n) − correction(n)
	

## Appendix C. Full Result Table

**Table A1:** R-peak detection performance evaluated in motion artifact regions only. *TP*: true positives, *FP*: false positives, *FN*: false negatives, *Se*: sensitivity, *PPV*: positive predictive value, *F1*: *F1* score. Config: C = coplanar, S = stacked. Env: A = Environment A (high interference), B = Environment B (low interference). Δ*F1* represents improvement in *F1* score after filtering. Empty cells indicate sessions excluded due to battery-related timing inconsistencies. Sessions marked with * (S5 Coplanar Env A, S6 Coplanar Env A) had gel reference electrode degradation during the third motion stage; recordings were truncated to the valid portion.

Subject/Config/Env			Raw Signal (Motion Only)						Filtered Signal (Motion Only)						Δ*F1*
			*TP*	*FP*	*FN*	*Se*	*PPV*	*F1*	*TP*	*FP*	*FN*	*Se*	*PPV*	*F1*	
S1	C	A	108	9	3	0.973	0.923	0.947	108	4	3	0.973	0.964	0.969	0.021
S1	C	B	88	7	2	0.978	0.926	0.951	88	3	2	0.978	0.967	0.972	0.021
S1	S	A	95	13	5	0.950	0.880	0.913	94	1	6	0.940	0.989	0.964	0.051
S1	S	B	95	13	4	0.960	0.880	0.918	94	10	5	0.949	0.904	0.926	0.008
S2	C	A	75	35	8	0.904	0.682	0.777	78	15	5	0.940	0.839	0.886	0.109
S2	C	B	74	61	11	0.871	0.548	0.673	74	40	11	0.871	0.649	0.744	0.071
S2	S	A	63	110	23	0.733	0.364	0.486	60	53	26	0.698	0.531	0.603	0.117
S2	S	B	72	66	14	0.837	0.522	0.643	81	47	5	0.942	0.633	0.757	0.114
S3	C	A	67	35	17	0.798	0.657	0.720	69	26	15	0.821	0.726	0.771	0.051
S3	C	B	69	58	15	0.821	0.543	0.654	72	30	12	0.857	0.706	0.774	0.120
S3	S	A	67	89	28	0.705	0.429	0.534	74	75	21	0.779	0.497	0.607	0.073
S3	S	B	62	87	32	0.660	0.416	0.510	74	61	20	0.787	0.548	0.646	0.136
S4	C	A	86	5	3	0.966	0.945	0.956	88	1	1	0.989	0.989	0.989	0.033
S4	C	B	59	11	5	0.922	0.843	0.881	55	3	9	0.859	0.948	0.902	0.021
S4	S	A	93	7	2	0.979	0.930	0.954	92	3	3	0.968	0.968	0.968	0.015
S4	S	B	68	39	8	0.895	0.636	0.743	69	15	7	0.908	0.821	0.863	0.119
* S5	C	A	64	14	9	0.877	0.821	0.848	65	15	8	0.890	0.812	0.850	0.002
S5	C	B	93	56	26	0.782	0.624	0.694	94	21	25	0.790	0.817	0.803	0.109
S5	S	A	-	-	-	-	-	-	-	-	-	-	-	-	-
S5	S	B	-	-	-	-	-	-	-	-	-	-	-	-	-
* S6	C	A	31	101	25	0.554	0.235	0.330	30	86	26	0.536	0.259	0.349	0.019
S6	C	B	41	133	35	0.539	0.236	0.328	42	112	34	0.553	0.273	0.365	0.037
S6	S	A	47	93	29	0.618	0.336	0.435	39	51	37	0.513	0.433	0.470	0.035
S6	S	B	46	129	40	0.535	0.263	0.352	43	84	43	0.500	0.339	0.404	0.051
S7	C	A	61	103	18	0.772	0.372	0.502	60	91	19	0.759	0.397	0.522	0.020
S7	C	B	-	-	-	-	-	-	-	-	-	-	-	-	-
S7	S	A	72	83	27	0.727	0.465	0.567	75	64	24	0.758	0.54	0.63	0.063
S7	S	B	-	-	-	-	-	-	-	-	-	-	-	-	-

**Table A2:** RMS amplitude in motion artifact regions before (*RMS*_raw_) and after (*RMS*_filt_) adaptive filtering, with percentage reduction (Δ*RMS*%). RMS values are in arbitrary units consistent with the ADC output.

	Coplanar, Env A			Coplanar, Env B			Stacked, Env A			Stacked, Env B		
	*RMS*_raw_	*RMS*_filt_	∆*RMS*%	*RMS*_raw_	*RMS*_filt_	∆*RMS*%	*RMS*_raw_	*RMS*_filt_	∆*RMS*%	*RMS*_raw_	*RMS*_filt_	∆*RMS*%
Subject 1	19.84	11.67	58.8	22.80	9.82	56.9	22.75	7.87	65.4	26.98	18.50	68.6
Subject 2	45.51	13.20	71.4	189.90	71.97	62.1	180.82	53.56	70.4	67.67	21.78	67.8
Subject 3	30.43	19.11	62.8	31.38	12.35	60.7	54.27	15.84	70.8	44.61	14.75	66.9
Subject 4	15.35	9.07	40.9	31.05	11.02	64.5	15.84	9.45	40.3	37.24	25.32	68.0
Subject 5	17.10	10.29	39.8	44.39	25.15	43.3	-	-	-	-	-	-
Subject 6	351.97	93.66	73.4	265.35	135.42	49.0	59.65	16.17	72.9	111.58	27.27	75.6
Subject 7	36.24	10.43	71.2	-	-	-	15.80	7.14	54.8	-	-	-

**Table A3:** R-peak detection performance evaluated across the entire recording (rest and motion regions combined). Column definitions as in Table A1. Δ*F1* values are smaller than in Table A1 because rest regions, where the algorithm does not apply correction, dilute the motion-region improvement.

Subject/Config/Env			Raw Signal (Whole Signal)						Filtered Signal (Whole Signal)						Δ*F1*
			*TP*	*FP*	*FN*	*Se*	*PPV*	*F1*	*TP*	*FP*	*FN*	*Se*	*PPV*	*F1*	
S1	C	A	187	9	6	0.969	0.954	0.961	187	4	6	0.969	0.979	0.974	0.013
S1	C	B	159	8	2	0.988	0.952	0.97	159	3	2	0.988	0.981	0.985	0.015
S1	S	A	137	13	6	0.958	0.913	0.935	136	1	7	0.951	0.993	0.971	0.036
S1	S	B	145	13	4	0.973	0.918	0.945	144	10	5	0.966	0.935	0.95	0.006
S2	C	A	123	35	8	0.939	0.778	0.851	126	15	5	0.962	0.894	0.926	0.075
S2	C	B	131	61	11	0.923	0.682	0.784	131	40	11	0.923	0.766	0.837	0.053
S2	S	A	118	111	25	0.825	0.515	0.634	109	53	34	0.762	0.673	0.715	0.080
S2	S	B	123	66	14	0.898	0.651	0.755	132	47	5	0.964	0.737	0.835	0.081
S3	C	A	133	35	18	0.881	0.792	0.834	135	26	16	0.894	0.839	0.865	0.032
S3	C	B	131	58	15	0.897	0.693	0.782	134	30	12	0.918	0.817	0.865	0.082
S3	S	A	136	90	29	0.824	0.602	0.696	143	76	22	0.867	0.653	0.745	0.049
S3	S	B	125	87	33	0.791	0.590	0.676	138	61	20	0.873	0.693	0.773	0.097
S4	C	A	156	5	3	0.981	0.969	0.975	158	1	1	0.994	0.994	0.994	0.019
S4	C	B	140	11	6	0.959	0.927	0.943	135	3	11	0.925	0.978	0.951	0.008
S4	S	A	156	8	4	0.975	0.951	0.963	154	4	6	0.963	0.975	0.969	0.006
S4	S	B	115	39	8	0.935	0.747	0.830	116	15	7	0.943	0.885	0.913	0.083
S5	C	A	121	15	10	0.924	0.890	0.906	123	16	8	0.939	0.885	0.911	0.005
S5	C	B	172	57	33	0.839	0.751	0.793	175	23	30	0.854	0.884	0.868	0.076
S5	S	A	-	-	-	-	-	-	-	-	-	-	-	-	-
S5	S	B	-	-	-	-	-	-	-	-	-	-	-	-	-
S6	C	A	76	106	27	0.738	0.418	0.533	76	90	27	0.738	0.458	0.565	0.032
S6	C	B	104	138	37	0.738	0.430	0.543	107	113	34	0.759	0.486	0.593	0.050
S6	S	A	101	97	31	0.765	0.510	0.612	91	52	41	0.689	0.636	0.662	0.050
S6	S	B	100	138	53	0.654	0.420	0.512	98	90	55	0.641	0.521	0.575	0.063
S7	C	A	134	112	21	0.865	0.545	0.668	134	97	21	0.865	0.580	0.694	0.026
S7	C	B	-	-	-	-	-	-	-	-	-	-	-	-	-
S7	S	A	134	87	32	0.807	0.606	0.693	138	67	28	0.831	0.673	0.744	0.051
S7	S	B	-	-	-	-	-	-	-	-	-	-	-	-	-
